# The impact of health promotion training on university students’ health perceptions, healthy lifestyle behaviors, and risky behaviors

**DOI:** 10.3389/fpsyg.2024.1407233

**Published:** 2024-12-13

**Authors:** Bahar Kefel¡ Çol, Ayşe Gümüşler Başaran, Burcu Genç Köse

**Affiliations:** ^1^Güneysu School of Physical Therapy and Rehabilitation, Recep Tayyip Erdoğan University, Rize, Türkiye; ^2^Faculty of Health Sciences, Recep Tayyip Erdoğan University, Rize, Türkiye

**Keywords:** healthy, lifestyle, smoking, suicide, antisocial behavior

## Abstract

**Introduction:**

University period is a critical developmental stage in which health-related behaviors that will be effective throughout life are acquired. This study aims to to evaluate the effect of health promotion education given touniversity students on health perception, health behaviors and risky behaviors, to investigate the impact of the demographic characteristics of the participants on these three variables, and to investigate the potential relationship between the three variables.

**Methods:**

It is a quasi-experimental study using a pre-post test design This study was conducted with 179 students. The Health Perception Scale, the Healthy Lifestyle Behaviors Scale, and the Risky Behaviors Scale were used to collect the data. A 7-week training program was implemented, and the post-training evaluation was made after 3 months. Percentage, mean, standard deviation, Mann–Whitney U, Kruskal-Wallis, Wilcoxon analysis, and Spearmen Correlation analysis were used to analyze the data.

**Results:**

After health promotion training, it was determined that health perception and healthy lifestyle behaviors increased and risky behaviors decreased. A significant negative relationship was found between health perception after training and antisocial behavior, suicidal tendencies and dropping out of school. A significant negative relationship was found between healthy lifestyle habits and antisocial behavior, suicidal tendencies and school dropout. A positive significant relationship was determined between health perception and healthy lifestyle behaviors. The results of this study provide evidence of the effectiveness of training interventions in improving healthy lifestyle behaviors and health perception and reducing risky behaviors.

**Discussion:**

The results support the planning of training programs to promote health on campuses. It also shows the effectiveness of training programs in preventing antisocial behavior, suicidal tendencies and school dropout.

## Introduction

The university years are a critical developmental period in which the individual develops the autonomy to leave home and make decisions, and gain health behaviors that will be effective throughout their life (Nawsherwan et al., 2021) and can lead to unhealthy outcomes for students (Nawsherwan et al., 2021; Guedes et al., 2024). University students are at risk of developing unhealthy lifestyle habits such as poor diet and lack of physical activity due to various factors such as financial conditions, academic pressures, lack of social support, forming new friendships, managing money (Marendić et al., 2024; Almoraie et al., 2024). Young adults attending higher education institutions living away from home are more likely to experience adverse changes in their eating habits (Palamutoglu et al., 2024), gain weight (Wilson et al., 2024; Du et al., 2023), become more physically inactive (Winpenny et al., 2020), experience higher levels of depression, anxiety and stress (Brett et al., 2023; Worsley et al., 2021). The university years are considered a period in which students tend to have unhealthy lifestyle behaviors and high rates of health-related risk factors (Whatnall et al., 2019). During this period, interventions are needed to gain positive health behaviors and reduce risky health behaviors, but intervention studies in this direction are limited (Hutchesson et al., 2022; Pfledderer et al., 2022).

Healthy lifestyle habits are those that people manage and incorporate into their everyday lives to improve their health (Zeng et al., 2021). According to the health promotion model, physical activity, stress management, nutrition, health responsibility, interpersonal relationships, and spiritual development are healthy lifestyle behaviors (Walker et al., 1987). Physical inactivity is a significant risk factor for, diabetes, dementia, coronary heart disease, stroke, and cancer (such as breast, colon) (Sivanantham et al., 2021; Friedenreich et al., 2021; Katzmarzyk et al., 2022). Most university students are not physically active (Brown et al., 2024; Amiri et al., 2019), and during their university years, there is a noticeable drop in the frequency of physical activity and exercise (Alkhateeb et al., 2019), which may continue in the following years (Kallio et al., 2020). Stress can damage all body systems, causing significant health problems (Zafar et al., 2021). Stress is a risk factor for chronic diseases such as cardiovascular disease (O'Connor et al., 2021), and type 1 diabetes (Ramani et al., 2020). Stress affects young individuals, particularly in college (Lian and Wallace, 2020; Islam et al., 2022). In general, university students do not eat regularly, skip breakfast and meals, (Alshdifat et al., 2024), prefer fast food (Ramesh et al., 2021), consume little daily fruit and vegetables, and have an imbalanced diet (Abdelhafez et al., 2020). Children and adolescents are more likely to be overweight or obese, and this trend persists throughout adulthood as a result of unhealthy diets (Woo et al., 2020; Ruiz et al., 2019). In addition to being overweight/obese, being underweight is also a common problem (Phelps et al., 2024). Health responsibility encourages healthy behaviors (e.g., exercise weight control) (Tabrizi et al., 2024). Studies show that university students’ health responsibility levels are generally low (Musić et al., 2021; Al-Momani, 2021; Hwang and Oh, 2020). People with strong and healthy interpersonal relationships tend to be healthier (Intiful et al., 2021). Healthy interpersonal relationships strengthen social support, promote good nutrition, increase physical activity, improve coping with adversity and stress, enhance personal development and control, and mediate health promotion (Xiang et al., 2024; Liu and Sun, 2023; Cao et al., 2023; Kim et al., 2021). In addition, interpersonal relationships at school effect the prevalence of suicidal ideation, school adaptation, smoking and school dropout (Baalmann et al., 2024; Nakano et al., 2022; Zhang et al., 2021; Karletsos et al., 2021; McCoy et al., 2020). Spiritual health has positive effects on physical, social, psychological health as well as other positive health outcomes such as health-related quality of life, coping skills, alleviating pain, ensuring a good attitude during traumatic situations or upsetting occurrences, less addiction or suicidal behaviors (Badnava et al., 2024; Maazallahi et al., 2021; Bożek et al., 2020; Mathad et al., 2019). A study found that the spiritual development of students was at a moderate level (Feizi et al., 2020). As previously indicated, students should be developed in physical activity, stress management, nutrition, health responsibility, interpersonal interactions, and spiritual growth during a challenging time like university. The other variable in the study, health perception, is defined as health-related feelings, thoughts, beliefs, expectations and prejudices that enable focusing on health, protecting, maintaining and improving health (Sınan et al., 2024; Oral and Cetinkaya, 2020; Souto et al., 2018). Protecting and promoting health requires a positive attitude toward health (Hwang and Oh, 2020). It is argued that a mutual interaction exists between health perception and health behaviors (de-Mateo-Silleras et al., 2019; Tunc et al., 2021). Healthy eating (Toral et al., 2021) and improved physical activity (de-Mateo-Silleras et al., 2019) have a positive effect on health perception, and health perception has a positive impact on sport participation (Lee et al., 2020). Health perception is reported to be low or moderate among university students (Tunc et al., 2021; Oral and Cetinkaya, 2020; de-Mateo-Silleras et al., 2019). To improve students’ perceptions of health inside the institution, it is crucial to spread educational initiatives.

Health-risk behaviors include those that jeopardize life, endanger health, raise the possibility of adverse physical, psychological, and social outcomes, and keep children from developing into mature, self-reliant individuals. Such behaviors may occur in the form of violence, tobacco, alcohol and substance use, suicidal tendency, school dropout, risky sexual intercourse, and negative eating habits (Shekari et al., 2020). The literature has citations that antisocial behaviors such as violence, aggression, crime (Younas et al., 2023; Ganson et al., 2022; Ye et al., 2022); smoking, alcohol, drug use (Sharareh et al., 2020; Htet et al., 2020; Shegute and Wasihun, 2021); suicidal tendency (Kaggwa et al., 2022); school dropout (Pınar, 2020) is at a high level (Shekari et al., 2020) among university students who make up a large part of the young population.

Antisocial behaviors, one of the risky behaviors, are behaviors that do not comply with socially accepted values and moral norms (Adams and Millie, 2021). It starts in early adolescence, peaks in late adolescence or early adulthood, and decreases in later life (van de Groep et al., 2023). Ongoing antisocial behavior can cause physical, occupational, educational, and mental health problems (Canino et al., 2022). As one of the risky behaviors, smoking causes chronic obstructive pulmonary disease (COPD), and chronic diseases such as cancer and heart disease (CDC, 2021). Worldwide tobacco use causes more than 7 million deaths annually (WHO, 2022). Although attempts to reduce smoking are effective, the rate of smoking among university students is high, and the university years are a critical time to start smoking (Barrington-Trimis et al., 2020; Samara et al., 2020). The prevalence of smoking among students ranges from 6.8 to 40% (Telayneh et al., 2021; Ahmed et al., 2020; Hassan et al., 2019). In a study, it was found that 30 per cent of university students were smokers, and smokers had significantly higher absenteeism rates and more academic warnings than non-smokers (Alqahtani et al., 2023). To prevent smoking initiation and support cessation, it is crucial to integrate smoking into health education, especially in higher education institutions.

Another risky behavior is suicidality, a worldwide public health issue (Haase et al., 2022). Suicide attempt is widespread among university students (Kaggwa et al., 2022; Owusu-Ansah et al., 2020; Mamun et al., 2020). Suicide is the fourth leading cause of death among 15–29-year-olds (WHO, 2023). Lifestyle behaviors such as smoking, alcohol use, and sedentary life are effective in increasing the risk of suicide (Kim et al., 2023; Berardelli et al., 2018).

The tendency for university dropout can also be considered a risky behavior and a significant global problem in developed and developing countries (Aldahmashi et al., 2021). It is a challenging situation that causes economic losses due to its consequences on the individual, family, and society (Sosu and Pheunpha, 2019; Niyogisubizo et al., 2022). School dropout is associated with health risk behaviors like smoking/alcohol, and drug use (Heradstveit et al., 2024; Hjarnaa et al., 2023; Ajith et al., 2022), being overweight/obese (Diaz-Serrano and Stoyanova, 2023) and stress (Pascoe et al., 2020), difficulties in coping with problems, bullying incidents (Cimene et al., 2023). Youth who drop out of school are more likely to experience socio-emotional difficulties, become delinquent, and engage in criminal behavior (Khurram et al., 2023). planning educational interventions to reduce the risk of dropping out is necessary.

After all these considerations, it is crucial for college students majoring in health-related fields to adopt healthy lifestyle practices, cut back on risky behavior, and enhance their positive perceptions of their health to be healthy individuals and role models for society.

The main aim of this study is to evaluate the effect of health promotion training given to university students on health perception, health behaviors and risky behaviors. The sub-objectives assess how the three variables are related to demographic characteristics and determine the relationship between health perception, health behaviors and risky behaviors.

## Methods

### Research design

The study is a quasi-experimental research conducted using group pre test -post test to evaluate the effect of the health promotion education program given to university students on healthy lifestyle behaviors, health perception and risky behaviors (de Vocht et al., 2021). The study population consisted of 251 first-year university students enrolled in the vocational school of health services and receiving associate degree education in health-related departments (anesthesia, first and emergency aid, medical laboratory and elderly care) in 2020. To reach the maximum sample size, when the incidence of the event was taken as 50%, the sample size for the study was calculated as 153 for p:0.50, t:0.05, d:0.05. The calculation process was performed using Raosoft Sample Size Calculation Program. The study was completed with 179 students who attended the training regularly, completed the questionnaires, and voluntarily agreed to participate. Research hypotheses.

*H1*: There is a significant difference between socio-demographic variables and healthy lifestyle behaviors.

*H2*: There is a significant difference between socio-demographic variables and health perception.

*H3*: There is a substantial difference between socio-demographic variables and risky behaviors.

*H4*: Health promotion training increases healthy lifestyle behaviors.

*H5*: Health promotion training increases health perception.

*H6*: Health promotion training reduces risky behaviors.

*H7*: There is a relationship between health perception and healthy lifestyle behaviors.

*H8*: There is a relationship between health perception and risky behaviors.

*H9*: There is a relationship between healthy lifestyle behaviors and risky behaviors.

### Data collection process

Data were collected using personal information form and scales. All students were invited to the study. The purpose of the study was explained to the students face to face, and it was demonstrated that participation in the study was voluntary and anonymous. The verbal consent of the students who volunteered to participate in the study was obtained and the personal information form and scales were shared. The data were collected by distributing the forms under observation in the classroom environment and asking the students to fill them out.

### Data collection tools

The data were collected using “The Personal Information Form”, “The Health Perception Scale”, “The Healthy Lifestyle Behaviors II” and “The Risky Behaviors Scale”.

*The researchers developed the Personal Information Form*; which consists of seven questions about gender, age, department, graduation, employment status, height, and weight (Annex I). *The “Health Perception Scale (HPS)”;* devised by Diamond et al. (2007), is a five-point Likert-type instrument designed initially in English (Diamond et al., 2007). The reliability and validity of its Turkish version were validated by Kadıoğlu and Yıldız (2012). The scale encompasses 15 items segmented into four sub-dimensions. Items 1, 5, 9, 10, 11, and 14 are positively framed, while items 2, 3, 4, 6, 7, 8, 12, 13, and 15 are constructed negatively. Positive items are scored as: “Strongly agree = 5,” “Agree = 4,” “Neutral = 3,” “Disagree = 2,” and “Strongly disagree = 1.” Negative items, on the other hand, are scored in reverse. The potential score on the scale spans from a minimum of 15 to a maximum of 75.

The scale delineates four sub-dimensions: “Control Center,” “Self-Awareness,” “Certainty,” and “Importance of Health.” The “Control Center” dimension is gauged by items 2, 3, 4, 12, and 13; the “Certainty” dimension by items 6, 7, 8 and 15; the “Importance of Health” dimension by items 1, 9 and 11; and the “Self-Awareness” dimension by items 5, 10 and 14. The Cronbach Alpha reliability coefficient of the scale was previously reported as 0.77 (Kadıoğlu and Yıldız, 2012). In this study, the total Crombach *α* values of the scale were determined as 0.70 (Annex II).

***The “Healthy Lifestyle Behaviors II (HLBS-II)”;*** The HLBS-II, developed by Walker et al. (1987), was revised by Walker and Hill-Polerecky (1996) and called “Healthy Lifestyle Behaviors II” (Walker and Hill-Polerecky, 1996). The reliability and validity of its Turkish version were validated by Bahar et al. (2008). The HLBS-II consists of 52 items and six sub-dimensions. Subdimensions are: 1. Spiritual development, 2. Health responsibility, 3. Physical activity, 4. Nutrition, 5. Interpersonal relations, and 6. Stress management. The “Health responsibility” dimension is gauged by items 3, 9, 15, 21, 27, 33, 39, 45 and 51; the “Physical activity,” dimension by items 4, 10, 16, 22, 28, 34, 40 and 46; the “Nutrition” dimension by items 2, 8, 14, 20, 26, 32, 38, 44 and 50; the “Interpersonal relations, dimension by items 1, 7, 13, 19, 25, 31, 37, 43, and 49; the “Stress management” dimension by items 5, 11, 17, 23, 29, 35, 41, and 47 and the “Spiritual development” dimension by items 6, 12, 18, 24, 30, 36, 42, 48, and 52. The overall score of the scale gives the score for healthy lifestyle behaviors. It is a 4-point Likert type scale. The lowest and the highest scores for the whole scale are 52 and 208. The Cronbach Alpha reliability coefficient of the scale was previously reported as 0.92 (Bahar et al., 2008). In this study, the total Crombach *α* values of the scale were determined to be 0.90 (Annex III). *The “Risky Behaviors Scale (RBS)”;* The scale was developed by Genctanırım (2014) to evaluate the Risky Behaviors of university students, and validity and reliability studies were conducted. It is a 5-point Likert-type scale with 60 items. It has seven sub-dimensions, including antisocial behaviors, alcohol, smoking, suicidal tendency, eating habits, school dropout, and drug use. The “Antisocial behaviors” dimension is gauged by items (1.–10.); the “Alcohol,” dimension by items (11.–19.); the “Smoking” dimension by items (20.–27.); the “Suicidal tendency,” dimension by items (28.–39.); the “Eating habits” dimension by items (40.–47.); the “School dropout” dimension by items (48.–51.); and the “Drug use” dimension by items (52.–60.). Since the dimensions of the scale are not related to each other, the total score of the scale is not calculated, and high scores indicate a high-risk level in that dimension. In this study, antisocial behaviors, smoking, suicidal tendency, and school dropout sub-dimensions were evaluated. The Cronbach Alpha reliability coefficient of the scale was previously reported to be between 0.64 and 0.92 (Genctanırım, 2014). In this study, the Crombach *α* values of the sub-dimensions were found to be 0.79 for antisocial behaviors, 0.92 for smoking, 0.94 for suicidal tendency, and 0.63 for school dropout (Annex IV).

### Intervention

The students were divided into four groups (anesthesia, first and emergency aid, medical laboratory and elderly care) according to the programs they studied at the university and the same training program was applied at different times. The intervention program was used for each group on a different day, in three sessions once a week for 7 weeks. The health perception scale evaluate students’ health perception before starting the training program, healthy lifestyle behaviors scale to assessed health behaviors, and the risky behavior scale was applied as a pre-test to evaluate the risky behaviors. In the training program, PowerPoint presentation, which is a computer-aided teaching program that strengthens the student’s learning processes and develops thinking skills such as analysis and synthesis, enhancing recall and interactive learning approach were used in coordination (Dewi et al., 2024; Twizeyimana et al., 2021). In interactive learning, the question-answer method, which is a method of making students think, drawing attention to important points and helping students find the answers, and the discussion method were used to direct students to think about a subject, to explain points that were not well understood and to reinforce previously given information (Bala et al., 2024; Verma et al., 2021; Tuma, 2021). In this training program, the following training topics were created, focusing on developing a positive health perception in the individual, regular physical activity/exercise, adequate and balanced nutrition, developing positive interpersonal relationships, effectively coping with stress and raising awareness about the harms of smoking. Three months after the training program was completed, post-test data were collected to evaluate the effectiveness of the training.

Training program; 1st week: The concept, dimensions, and determinants of health, health protection and health, 2nd week: Healthy lifestyle behaviors, 3rd week: Physical activity\exercise, 4th week: Nutrition, 5th week: Interpersonal relationships and social support, 6th week: Stress management, 7th week: Smoking.

### Data analysis

SPSS 22 package program was used for statistical analyses of the data, and descriptive data were expressed as percentages, mean and standard deviation. Mann–Whitney U, Kruskal-Wallis, and Wilcoxon analyses were used to statistically analyze of quantitative data. Spearman correlation analysis was used to evaluate the relationship between variables. The significance value was accepted as *p* < 0.05. In the correlation analysis, 0–0.19 was considered as no correlation, 0.20–0.39 as a weak correlation, 0.40–0.69 as a moderate correlation, 0.70–0.89 as a strong correlation, 0.90–1.00 as a robust correlation. The significance value was taken as *p* < 0.05 (Alpar, 2016).

### Ethical considerations

This study was approved by the Non-Interventional Clinical Research Ethics Committee of the Faculty of Medicine of the XX-XX-XX University (Approval no. 40465587-102.01-96).

## Results

Of the students in the study, 85.5% were women, and 14.5% were men with a mean age of 20.10 ± 1.78 years. 30.7, 29.6, 26.8, and 12.8% of the students were in the Anesthesia Technician, First and Emergency Aid, Medical Laboratory, and Elderly Care Departments, respectively. 78.8% were vocational high school graduates, 2.8% were working, and 30.7% smoked. 28.2% of those who smoked were women, and 46.2% were men. The mean BMI of the students was 21.62 ± 2.98, 12.3% were overweight, 1.1% were obese, and 14.5% were underweight. The rates of being overweight/obese and underweight were 10.5–15.7% in women and 7.7–30.8% in men.

The pre-training and post-training mean scores of the Health Perception Scale (HPS) were 52.98 ± 5.96 and 54.96 ± 6.51, respectively. The pre-training and post-training mean scores of the healthy lifestyle scale were 127.87 ± 17.95 and 142.12 ± 22.63, respectively. The mean scores of the students on the Health Perception Scale, Healthy Lifestyle Scale and its sub-dimensions, and Risky Behaviors Scale are shown in Table 1.

**Table 1:** Pre-post training health perception scale, risky behaviors scale, and healthy lifestyle behaviors scale scores (*N* = 179).

	Pre-training			Post-training		
The Health Perception Scale Subdimensions	n	X ± SD	Min–Max	n	X ± SD	Min–Max
Health Perception Scale Total	179	52.84 ± 5.96	36–75	179	54.96 ± 6.51	40–75
Center of control	179	17.28 ± 3.43	7–25	179	17.87 ± 3.47	7–25
Certainly	179	12.78 ± 3.02	4–20	179	13.46 ± 3.14	4–20
Importance of health	179	11.53 ± 1.81	5–15	179	11.74 ± 1.86	6–15
Self-awareness	179	11.39 ± 1.89	3–15	179	11.89 ± 1.90	6–15

The Healthy Lifestyle Behaviors Scale Subdimensions	*n*	X ± SD	Min–Max	*n*	X. ± SD	Min–Max
The Healthy Lifestyle Behaviors Scale Total	179	127.87 ± 17.95	92–198	179	142.12 ± 22.63	84–200
Health responsibility	179	19.92 ± 4.56	9–34	179	23.24 ± 5.09	12–35
Physical activity	179	16.44 ± 4.54	8–31	179	19.87 ± 4.84	8–32
Nutrition	179	19.53 ± 4.05	10–33	179	22.49 ± 4.53	14–34
Spiritual Development	179	26.82 ± 4.01	16–36	179	28.02 ± 4.54	13–36
Interpersonal relationships	179	25.74 ± 4.09	15–36	179	26.91 ± 4.31	14–36
Stress management	179	19.42 ± 3.54	10–29	179	21.60 ± 3.57	13–32

Risky Behaviors Scale Subdimensions	*n*	X ± SD	Min–Max	*n*	X. ± SD	Min–Max
Antisocial behaviors	179	17.60 ± 5.46	10–43	179	16.31 ± 5.11	10–35
Smoking	179	14.80 ± 8.08	8–40	179	13.62 ± 7.46	8–38
Suicidal tendency	179	23.82 ± 10.27	12–58	179	20.90 ± 6.68	12–55
School dropout	179	8.34 ± 3.44	4–18	179	7.73 ± 3.42	4–17

No significant difference was found in the HPS total score and subscales according to gender, department and BMI (*p* = 0.386, *p* = 0.415, *p* = 0.265) (Table 2). There were no significant differences in the Healthy Lifestyle Behaviors scale (HLBS) and its sub-dimensions according to the department, type of high school, and BMI (*p >* 0.005). Gender created a significant difference in terms of the physical activity and interpersonal relationships sub-dimensions of the HLBS and total score, which was found to be higher in men (*p* < 0.001, *p* = 0.014, *p* = 0.014) (Table 2). After the training, there was a significant difference in physical activity in men (*RM =* 113.15) than women (*RM =* 86.07) (*MWU =* 1387.0, *z =* −2.470, *p =* 0.014). Gender made a significant difference in the sub-dimensions of smoking and school dropout on the risky behaviors scale and was found to be higher in men (*p* < 0.001, *p* = 0.003). School dropout was significantly higher in Medical Laboratory and Elderly Care departments than in the First and Emergency Aid departments (*p =* 0.035) Smoking was found to be significantly higher in overweight students than in underweight students (*p =* 0.047) (Table 2). In the analysis of the risky behaviors scale after the training, antisocial behaviors (*MWU =* 1372.0, *p =* 0.011), smoking (*MWU =* 1016.5, *p <* 0.0001), and school dropout (*MWU =* 1501.0, *p* = 0.044) dimensions were significantly higher in men Smoking was found to be significantly higher in overweight (*RM =* 111.82) than in underweight (*RM =* 66.25) students (*X^2^ =* 10.085, *p =* 0.018).

**Table 2:** Evaluation of the healthy lifestyle behaviors scale and its subdimensions and risky behaviors scale subdimensions according to sociodemographic characteristics (*N* = 179).

Independent variables		Health perception scale total	Center of control	Certainly	Importance of health	Self-awareness
		X ± SS	Rank mean	X ± SS	Rank mean	Rank mean
Gender	Women	53.14 ± 5.69	91.82	12.78 ± 3.02	90.11	91.16
	Men	52.04 ± 7.42	79.29	12.81 ± 3.06	89.35	83.17
		*t* = 0.868	U = 1710.5	t = −0.047	U = 1972.0	U = 1811.5
		*p* = 0.386	*p* = 0.252	*p* = 0.963	*p* = 0.944	*p* = 0.460
Department	Anesthesia	52.16 ± 6.53	83.62	12.42 ± 3.41	80.45	98.15
	First and emergency aid	53.40 ± 4.73	94.01	13.02 ± 2.80	92.73	80.84
	Medical laboratory	52.81 ± 6.87	93.59	12.73 ± 2.88	90.75	88.08
	Elderly care	54.30 ± 5.02	88.52	13.22 ± 2.86	105.00	95.63
		*F* = 0.818	KWX^2^ = 1.415	*F* = 0.535	KWX^2^ = 4.097	KWX^2^ = 3.460
		*p* = 0.415	*p* = 0.702	*p* = 0.659	*p* = 0.251	*p* = 0.326
BMI	Underweight	51.62 ± 7.27	83.58	13.04 ± 2.62	85.81	70.29
	Normal	53.38 ± 5.92	90.24	12.78 ± 3.21	94.25	94.42
	Overweight	52.77 ± 4.20	98.39	12.68 ± 2.24	74.89	89.05
	Obese	47.00 ± 1.41	65.50	10.50 ± 2.12	36.75	71.50
		*F* = 1.335	KWX^2^ = 1.440	*F* = 0.447	KWX^2^ = 5.203	KWX^2^ = 5.124
		*p* = 0.265	*p* = 0.696	*p* = 0.720	*p* = 0.158	*p* = 0.163

Independent variables		The Healthy Lifestyle Behaviors Scale						
		Health responsibility	Physical activity	Nutrition	Spiritual development	Interpersonal relationships	Stress management	HLBS scale total
		Rank mean	Rank mean	Rank mean	Rank mean	Rank mean	Rank mean	Rank mean
Gender	Women	89.27	83.10	87.81	86.89	86.08	88.77	86.09
	Men	94.29	130.62	102.90	108.29	113.10	97.25	113.00
	MWU	1877.50	933.00	1653.50	1513.50	1388.50	1800.50	1391.00
	*p*	0.647	<0.001	0.168	0.051	0.014	0.438	0.014
Department	Anesthesia	92.47	91.85	86.50	91.94	84.02	92.41	89.46
	First and emergency aid	87.09	94.87	95.67	96.32	92.73	90.28	93.60
	Medical laboratory	84.13	83.86	84.10	76.92	87.97	87.33	81.05
	Elderly care	103.04	87.15	97.61	98.11	102.26	89.15	101.65
	KWX^2^	2.382	1.288	2.018	4.517	2.256	0.256	2.858
	*p*	0.497	0.732	0.569	0.211	0.521	0.968	0.414
BMI	Underweight	93.15	80.71	73.83	82.40	87.56	86.75	84.42
	Normal	91.91	93.01	93.34	92.14	90.31	92.15	92.83
	Overweight	80.57	83.16	91.27	90.14	95.02	81.70	83.84
	Obese	29.25	92.00	70.50	49.00	46.50	85.00	47.75
	KWX^2^	3.775	1.666	3.393	2.044	1.689	0.914	2.328
	*p*	0.287	0.645	0.335	0.563	0.639	0.822	0.507

Independent variables		Risky Behaviors Scale			
		Antisocial behaviors	Smoking	Suicidal tendency	School dropout
		Rank mean	Rank mean	Rank mean	Rank mean
Gender	Women	87.30	83.81	90.82	85.35
	Men	105.90	126.42	85.17	117.38
	MWU	1575.50	1042.00	1863.50	1277.00
	*p*	0.090	0.000	0.607	0.003
Department	Anesthesia	86.65	96.30	91.93	82.41
	First and emergency aid	80.02	88.16	85.25	79.45
	Medical laboratory	98.77	89.34	95.96	102.20
	Elderly care	102.70	80.54	83.91	107.00
	KWX^2^	4.891	1.676	1.478	8.600
	*p*	0.173	0.642	0.687	0.035
BMI	Underweight	77.81	68.04	106.15	77.15
	Normal	92.59	91.67	86.09	91.85
	Overweight	90.00	108.14	96.16	98.50
	Obese	81.50	68.25	64.50	44.25
	KWX^2^	1.826	7.958	4.067	3.954
	*p*	0.609	0.047	0.254	0.266

The health perception scale score of the students increased significantly after the training (*p* < 0.001). The comparison of the HPS scores before and after the training is shown in Table 3.

**Table 3:** Comparison of the pre-and post-training HPS, HLBS total and subdimension scores (*N* = 179).

	*n*	Pre-training	Post-training	Difference	*t*	*p*
HPS	179	52.84 ± 5.96	54.96 ± 6.51	−1.978 ± 6.26	−4.227	<0.001

HLBS total score post-test- pre-test	*n*	Mean rank	Rank total	*z*	*p*
Negative Rank	35	52.90	1851.50	−8.602	<0.001
Positive Rank	138	96.65	13199.50		
Equal	6	–	–		
Health responsibility Post-test- pre-test					
Negative Rank	30	58.32	1749.50	−8.005	<0.001
Positive Rank	130	85.62	11130.50		
Equal	19	–	–		
Physical activity Post-test- pre-test					
Negative Rank	33	45.94	1516.00	−8.805	<0.001
Positive Rank	134	99.37	12512.00		
Equal	12	–	–		
Nutrition Post-test- pre-test					
Negative Rank	34	56.60	1924.50	−8.030	<0.001
Positive Rank	131	88.95	770.50		
Equal	14	–	–		
Spiritual Development Post-test-pre-test					
Negative Rank	55	68.42	3763.00	−3.888	<0.001
Positive Rank	98	81.82	8018.00		
Equal	26	–	–		
Interpersonal relationships Post-test- pre-test					
Negative Rank	62	75.22	4663.50	−3.668	<0.001
Positive Rank	104	88.44	9197.50		
Equal	13	–	–		
Stress management Post-test- pre-test					
Negative Rank	38	59.84	2274.00	−7.125	<0.001
Positive Rank	122	86.93	10606.00		
Equal	19	–	–		

HPS, The Health Perception Scale; HLBS, The Healthy Lifestyle Behaviors.

After the training, it was found that 138 students increased their HLBS scores and the difference between the pre-and-post intervention scores was significant (*p* < 0.001). The effect of the training on the HLBS created a substantial difference in total and all sub-dimensions (*p* < 0.001), and the rank sums of the difference scores had an increasing effect on the total and sub-dimension scores of the HLBS (Table 3).

The effect of the training on the RBS was examined, and a significant difference was detected between the pre-and post-training scores in the sub-dimensions of antisocial behavior, smoking, suicidal tendency, and school dropout (*p* < 0.001, *p* < 0.001, *p* < 0.001, *p* < 0.001, *p* = 0.005). Considering the rank sums of the difference scores, it can be said that the training effectively reduced antisocial behavior, smoking, suicidal tendencies, and school dropout scores (Table 4).

**Table 4:** Comparison of students’ pre-and post-training RBS scores (*N* = 179).

Antisocial behaviors post-test- pre-test	*n*	Mean rank	Rank total	*z*	*p*
Negative Rank	103	78.99	8136.00	−3.573	<0.001
Positive Rank	53	77.55	4110.00		
Equal	23	–	–		
Smoking Post-test- pre-test					
Negative Rank	93	69.87	6498.00	−3.839	<0.001
Positive Rank	44	67.16	2955.00		
Equal	42	–	–		
Suicidal tendency Post-test- pre-test					
Negative Rank	104	90.97	9461.00	−4.086	<0.001
Positive Rank	62	70.97	4400.00		
Equal	13	–	–		
School dropout Post-test- pre-test					
Negative Rank	90	77.40	6966.50	−2.803	0.005
Positive Rank	58	70.00	4060.00		
Equal	31	–	–		

In the study, the change in the total and subscale scores of the students after the training was evaluated. If the change in the scores of HPS and HLBS scales after the training was in the direction of increase, it was accepted that the training had a positive effect on the students. In the risky behaviors scale, if there was a decrease in the score after the training, it was accepted as a positive effect. After the training, the percentage of students whose scores increased on the HPS scale was 61.5 and 77.1% in the HLBS total. In the risky behaviors scale, the rate of students whose risk decreased was 57.5% for antisocial behavior, 52% for smoking, 58.1% for suicide and 50.3% for school dropout. The percentages of positive change in the total and sub-dimensions of the scale after the training are shown in Table 5.

**Table 5:** Positive change in scale scores after training percentage of students (*n* = 179).

	*n*	%
The Health Perception Scale Subdimensions		
Health Perception Scale Total	110	61.5
Center of control	89	49.7
Certainly	93	52.0
Importance of health	82	45.8
Self-awareness	179	100
The Healthy Lifestyle Behaviors Scale Subdimensions		
The Healthy Lifestyle Behaviors Scale Total	138	77.1
Health responsibility	130	72.6
Physical activity	134	74.9
Nutrition	131	73.2
Spiritual Development	98	54.7
Interpersonal relationships	104	58.1
Stress management	122	68.2
Risky Behaviors Scale Subdimensions		
Antisocial behaviors	103	57.5
Smoking	93	52.0
Suicidal tendency	104	58.1
School dropout	90	50.3

In the pre-training Spearman correlation analysis, a weak positive correlation was found between BMI and smoking (*r =* 0.214, *p* = 0.004), a weak positive correlation between HPS and HLBS (*r =* 0.398, *p* < 0.001), and weak negative correlations between the subdimensions of antisocial behavior, suicidal tendency, and school dropout in RBS (*r = −*0.263, *p <* 0.001; *r = −*0.308, *p < 0.*001, *r = −*0.197, *p =* 0.008). There were weak significant negative correlations between HLBS and RBS sub-dimensions of antisocial behavior, suicidal tendency, and school dropout (*r = −*0.219, *p =* 0.003; *r = −*0.381, *p =* 0.000; *r = −*0.229, *p =* 0.002). A positive, weak, or moderately significant relationship was found between the subdimensions of the RBS. The post-training correlation analysis is shown in Table 6.

**Table 6:** Post-training correlation analysis of the sub-dimensions of the HPS, HLBS, RBS (*N* = 179).

		BMI	HPS total score	HLBS total score	Antisocial behavior	Smoking	Suicidal tendency	School dropout
Age	r	0.037	−0.014	0.007	−0.002	−0.130	0.082	0.017
	*p*	0.621	0.850	0.923	0.977	0.083	0.276	0.819
BMI	r		−0.066	−0.015	0.099	0.214	0.000	0.016
	*p*	1	0.381	0.839	0.186	0.004	>0.999	0.833
HPS total score	r		1	0.398	−0.263	−0.076	−0.308	−0.197
	*p*			0.000	0.000	0.312	0.000	0.008
HLBS total score	r			1	−0.219	−0.113	−0.381	−0.229
	*p*				0.003	0.132	0.000	0.002

HLBS, The Healthy Lifestyle Behaviors; BMI, Body Mass Index; HPS, The Health Perception Scale.

## Discussion

Most research in the literature examining university students’ healthy lifestyles is descriptive studies. It is reported that the healthy lifestyles of university or college students studying in health-related departments are moderate (Ajrash and Al-Abedi, 2024; Alothman et al., 2024; Baykal et al., 2022;, Fashafsheh et al., 2021; Gilan et al., 2021). Likewise, this study found healthy lifestyle behaviors to be moderate. Various studies show that university students should adopt healthy lifestyles, which requires intervention studies. Encouraging positive results were found in intervention studies conducted to improve a healthy lifestyle with education (Masini et al., 2024; Ricci et al., 2022; Solhi et al., 2020; Liu et al., 2019). Our study observed a significant increase in healthy lifestyle behaviors after the training intervention. The HLBS scale’s physical activity sub-dimension showed a substantial gender difference in the overall score, with men scoring higher, consistent with the literature (McCarthy and Warne, 2022; Mayo et al., 2020).

A study with university students found that 47.6% sat for at least 8 h daily (Edelmann et al., 2022). The prevalence of physically inactive university students ranges from 14.5 to 77.6% (Verma et al., 2022; Ndupu et al., 2023; Santana et al., 2023; Edelmann et al., 2022). Studies emphasized that there was a significant decrease in the rate of physical activity at university compared to previous school years (Wilson et al., 2021; Senarath et al., 2021; Alkhateeb et al., 2019), and that decrease continues in the following years (Kallio et al., 2020; MKID et al., 2021). A significant number of students studying in health-related departments such as physiotherapy, health, medicine, dentistry, nursing, and paramedic were not physically active, which is parallel with this study (Eymirli et al., 2024; Dmitruk and Hołub, 2024; Schramlová et al., 2024; Alhammad et al., 2023; Cavus et al., 2020). Students studying in health departments had the lowest physical activity score among the the HLBS sub-dimensions (Gilan et al., 2021; Ghorabi et al., 2021). The significant increase in physical activity rate after the training provided in this study is similar to other studies (Casimiro-Andújar et al., 2023; Sharry and Timmins, 2016). However, the intensity of the education programs, such as theoretical, clinical, and field visits, may be why this rise was not at the expected level. Time is reported to be an inhibiting factor (Brown et al., 2024; Alhammad et al., 2023), and physical activity is seen as a leisure time activity without being integrated into daily life.

Many studies have emphasized that young adults experience stress, especially during their university years (), and perceived stress is at a high level in most university students (Kamruzzaman et al., 2024; Alkhawaldeh et al., 2023; Amanvermez et al., 2023). Among the causes of stress in university students, there are generally academic concerns, balancing work and life, health, living away from home, relationships with their environment, personal life, economic problems, and concerns about the future (Khademian et al., 2021; Karyotaki et al., 2020). In this study, the level of stress management was low but increased after the training. Similarly, the literature shows that a stress management program is an effective strategy to help students exposed to stress (Gulnar et al., 2024; Akman et al., 2022; Hsu and Goldsmith, 2021). Therefore, it is recommended that stress management programs be implemented at universities.

University students generally do not have regular and balanced eating habits, compliance with the Mediterranean diet is low, do not eat breakfast regularly, consume insufficient fruits and vegetables, consume fried foods, and prefer high-fat, high-sugar, and high-calorie foods like fast food (Puente-Hidalgo et al., 2024; Bayomy et al., 2024; Benaich et al., 2021) Poor eating habits acquired during this period increase the risk of overweight/obesity and chronic diseases (Haider et al., 2024; Mahfouz et al., 2024; Manchester, 2020). The overweight/obesity rates of university students vary between 17.7 and 27.8% (Pitil and Ghazali, 2022; Ilić et al., 2024; Rotich et al., 2023). In health science students, being overweight/obese ranged between 34.4 and 40.0%, higher than the general university population (Makkawy et al., 2021; Rabanales-Sotos et al., 2020). This may be due to living alone, academic stress, high theoretical/clinical course hours (Almoraie et al., 2024; Ramón-Arbués et al., 2021). In this study, the rate of being overweight/obese (13.4%) was lower than in other studies. This is thought to be because, similar to other studies (Muscogiuri et al., 2024; McCarthy and Warne, 2022; Kim and Shin, 2020), the rate of being overweight/obese in female students (10.5%) was lower than in male students (30.8%) and the fact that the number of female students participating in the study (85.5%) was higher than that of males.

Like being overweight/obese, being underweight is a significant nutritional problem for university students. In this study, the rate of being underweight in students was found to be 14.5%. The literature supports this study and shows that the underweight rate of students is between 4.6 and 27.1% (Ghazawy et al., 2022; Ndung'u et al., 2024; Nagashima et al., 2024; Ahmad et al., 2023; Irfan et al., 2019). Women are at greater risk of being underweight than men due to biological, socioeconomic and cultural factors (Joh et al., 2024; Ikoona et al., 2023; Zhang et al., 2019). Similarly, in this study, the rate of being underweight was higher in women (15.7%) than in men (7.7%). This may be due to women students’ negative body image perception (Murofushi et al., 2023; Radwan et al., 2019). These results reveal the importance of organizing health promotion programs on campuses to highlight healthy body weight in students.

Our study demonstrated that students had low scores in the nutrition sub-dimension of the HLBS. Studies support this finding and show that students’ level of compliance with recommendations regarding healthy eating habits is insufficient (Castro-Cuesta et al., 2023; Sánchez-Ojeda et al., 2022; Assaf et al., 2019; Almutairi et al., 2018). Most students who study in health-related fields do not adhere to a strict diet, do not view their food choices as healthy, and wish to pay more attention to their health (Assaf et al., 2019). There was a statistically significant increase in the nutrition score after the health promotion training given to the students. Similar to this study, previous studies have found that nutritional training provided to university students was influential in developing healthy eating habits (López-Moreno et al., 2023). Luszczynska and Haynes (2009) explained that interventions designed to increase fruit and vegetable consumption among health-related department students can be effective. In light of these data, it can be said that students need training interventions to gain healthy eating habits.

In this study, students’ mean health responsibility scores were evaluated at a low level, which is consistent with the results of other studies (Musić et al., 2021; Al-Momani, 2021; Hwang and Oh, 2020; Almutairi et al., 2018). This may be because individuals at this age are generally healthy and, therefore, do not consider it necessary to pay attention to health responsibility. However, students should learn health responsibility throughout the university years, both for health and as health professionals after graduation, serving role models for people and directing them toward healthy practices. Health practitioners may be less inclined than others to advise their patients to adopt good living habits if they do not participate in healthy lifestyle behaviors (Egger et al., 2017; Belfrage et al., 2018). In this study, students’ health responsibilities increased significantly after the training. Consistent with the results of this study, in previous studies, training interventions improved students’ health responsibility (Solhi et al., 2020; Coban et al., 2017).

Positive interpersonal connections boost social and interpersonal support, help people deal with challenges and stress, help them obtain positive life experiences, and encourage personal growth, all of which contribute to health improvement (Dinis et al., 2019; Young et al., 2019). The current study’s results revealed that the students had a moderate level of interpersonal relations and that the health promotion training significantly increased the interpersonal relationship level of the students.

The highest mean scores of the students were found to be in spiritual growth, which is consistent with previous studies (Al-Momani, 2021; Fashafsheh et al., 2021). Studies emphasise a relationship between spiritual well-being, health habits, psychological discomfort, anger, sadness, and other unpleasant emotions (Bożek et al., 2020; Leung and Pong, 2021). At the same time, the literature emphasises that there is a relationship between spirituality and smoking cessation and that it has a protective role against participation in antisocial behaviors and suicidal behaviors (Azmi et al., 2021; Okwudili et al., 2020; Andrade et al., 2020). However, despite this study’s high level of spiritual well-being, no relationship was found between spiritual well-being and health perception and risky behaviors. A positive perception of health is essential for health promotion (Hwang and Oh, 2020). Research on university students has shown that students have low (Oral and Cetinkaya, 2020) and moderate health perception (Tunc et al., 2021). In our study, the students had a moderate level of health perception, but it increased significantly after health promotion training, parallel with the literature (Erenoglu et al., 2019; Cass et al., 2021). Health perception scores and health behaviors affect each other (Tunc et al., 2021; Toral et al., 2021; de-Mateo-Silleras et al., 2019). This research confirms previous research and shows a link between healthy lifestyle choices and beliefs about one’s health.

Research shows that both the tendency to show antisocial behavior (aggression, violence, bullying) and exposure to antisocial behaviors is significantly prevalent (Frías Armenta and Corral-Frías, 2021; Grant et al., 2016; Jeffrey et al., 2022). Therefore, effective intervention strategies are also necessary for the prevention of antisocial behaviors (Villafuerte-Díaz et al., 2024; Martínez-Otero and Gaeta, 2022; Davletbaeva et al., 2021). In this study, there was a significant decrease in students’ antisocial behaviors after health promotion training, and a significant negative relationship was found between health perception and healthy lifestyle behaviors and antisocial behavior after the training. Our findings suggest that healthy lifestyle activities and improved post-training health perception may help university students experience less antisocial behavior. Similar to the literature, our results indicate that antisocial behavior is associated with suicidal behavior (McCloskey and Ammerman, 2018) and school dropout (Vadivel et al., 2023; Gubbels et al., 2019) and revealed a relationship between antisocial behavior and smoking (Weiss et al., 2019).

Although tobacco control is considered the most important goal for public health, and many control policies have been implemented to combat smoking, it remains the most significant preventable cause of disease and premature death (Dai et al., 2022; Bafunno et al., 2020). The university period is risky in terms of starting/continuing smoking due to exposure to peers who smoke and social, emotional, and educational challenges (Alkhalaf et al., 2021; Ahmed et al., 2020). The prevalence of smoking among university students ranges from 17 to 32.6% (Samara et al., 2020; Habbash et al., 2023; Oguz et al., 2018; Sinnathamby et al., 2023; Ahmed et al., 2020). Similar to the literature, smoking was found to be 30.7% in this study. Men smoking is significantly higher than women smoking (Telayneh et al., 2021). Smoking prevalence among university students worldwide, including students studying in health-related fields, is alarming. It was found that smoking decreased significantly after training. Prospective healthcare workers should be encouraged to have healthy attitudes regarding smoking and quitting since they will eventually be community leaders and role models for lowering the smoking rate.

Suicidal tendency is reported to be high among university students (Kabir et al., 2024; Kabbash et al., 2023; Crispim et al., 2021; Wu et al., 2021). Suicidal tendencies in our study were at a low level and significantly decreased with the healthy lifestyle behaviors training like another relevant study (Engin et al., 2012). Modifiable health risk factors like a sedentary lifestyle (Li et al., 2021; Silva et al., 2020), skipping breakfast, drinking carbonated drinks (Michael et al., 2020; Berg et al., 2020), smoking drinks (Berardelli et al., 2018; Dasagi et al., 2021; Waters et al., 2021), and poor health perception (Isaac et al., 2018) are associated with suicidal behaviors (Zhan et al., 2024; Kim and Seo, 2023). In this study, a significant negative relationship was found between healthy lifestyle behaviors and health perception and suicidality, and a significant positive relationship was found between smoking and suicidal tendency.

Similar to earlier research on the subject, our study found that male students had a greater probability of dropping out of school (Zengin, 2021), which may be because, in traditional societies, the male gender is supported to behave more independently and freely. A significant positive relationship was found between school dropout and antisocial behaviors, smoking, and suicidal tendencies, and a significant negative relationship was found between health perception and healthy lifestyle behaviors in our study. In similar studies, dropouts were more likely to encounter individual negative behaviors (arrest, illegal substance use, poor health) than graduates (Dennison, 2022; Bae, 2020; Valkov, 2018; Lansford et al., 2016) and had a higher risk of smoking (Desai et al., 2019). Other studies show that obesity (Lanza and Huang, 2015), smoking (Svansdottir et al., 2015), alcohol use (Hjarnaa et al., 2023; Fernández-Suárez et al., 2016), anti-social behavior/cognitions, criminal behavior (Ward et al., 2021; Gubbels et al., 2019), violence (Montes and Mendes, 2021) and exposure to bullying (Bernardo et al., 2020) are risk factors for school dropout.

## Limitations

This study included only students from the vocational school of health services, which is not representative of the higher education population. The results obtained after the training provide data on the effect of short-term training. Planning new studies that require follow-up to understand the long-term impact is recommended. The fact that the data of this study were obtained through students’ self-reports can also be considered a limitation.

## Conclusion

Although it is often believed that students studying in health departments exhibit good lifestyle habits, including taking charge of their health, eating a sufficient and balanced diet, exercising frequently, and managing stress efficiently, research reveals that this is not always the case. In this study, students studying health were found to have a relatively weak health-promoting lifestyle. Deficient scores were found in physical activity, stress management, nutrition, and health responsibility subdimensions. In addition, women were in the risk group for physical activity, interpersonal relationships, and healthy lifestyle behaviors. The health perception of the students was found to be at a moderate level. In terms of risky behaviors, it was found that students were more likely than average to engage in antisocial behaviors, smoking, suicidal tendencies, and school dropout. Men students were more likely than women students to engage in these risky behaviors, and overweight students were more likely to smoke. These findings indicate that students need special attention to health-promoting behaviors. The results of this study provide evidence for the effectiveness of educational interventions in reducing risky behaviors while promoting healthy lifestyle habits and improving health perception.

## Data Availability

The raw data supporting the conclusions of this article will be made available by the authors, without undue reservation.

## References

[ref1] Abdelhafez A. I. Akhter F. Alsultan A. A. Jalal S. M. Ali A. (2020). Dietary practices and barriers to adherence to healthy eating among King Faisal University students. Int. J. Environ. Res. Public Health 17:8945. doi: 10.3390/ijerph17238945 33271893 PMC7731134

[ref2] Adams J. Millie A. (2021). Everyday moral judgements of anti-social behaviour. Crime Prev. Community Saf. 23, 56–68. doi: 10.1057/s41300-020-00106-6

[ref3] Ahmad S. N. F. F. Salehuddin N. S. Ahmad Sharoni S. K. Fauzi R. Buhari S. S. Nor M.. (2023). Weight management behaviors among students in a public university. Malays. J. Nurs. 14, 67–75. doi: 10.31674/mjn.2023.v14i03.008

[ref4] Ahmed M. S. Sayeed A. Jahan I. Dewan M. F. Mali S. K. (2020). Prevalence and factors associated with cigarette smoking among resident university students: a crosssectional study from Bangladesh. Popul. Med. 2, 1–6. doi: 10.18332/popmed/118250

[ref5] Ajith A. Temmen C. Haynie D. Choi K. (2022). Association between adolescent smoking and subsequent college completion by parent education: a national longitudinal study. Drug Alcohol Depend. 233:109360. doi: 10.1016/j.drugalcdep.2022.109360, PMID: 35228079 PMC8957578

[ref6] Ajrash K. A. Al-Abedi G. A. (2024). Healthy behaviors between medical and non-medical university students. Bahrain Med. Bull. 46, 1848–1851.

[ref7] Akman O. Yıldırım D. Oksuz S. (2022). The effect of mindful breathing exercise on stress levels of nursing first year students before first clinical experience. Gevher Nesıbe J. Med. Health Scı. 5, 1–8. doi: 10.46648/gnj.26

[ref8] Aldahmashi T. Algholaiqa T. Alrajhi Z. Althunayan T. Anjum I. Almuqbil B. (2021). A case-control study on personal and academic determinants of dropout among health profession students. High. Educ. Stud. 11, 120–126. doi: 10.5539/hes.v11n2p120

[ref9] Alhammad S. A. Almutairi F. M. Bajsair A. S. Alghamdi A. S. Algarni F. S. Aldaihan M. M.. (2023). Physical activity levels among undergraduate students at the College of Applied Medical Sciences, King Saud University, Riyadh: a prevalence study. Medicine (Baltimore) 102:e36386. doi: 10.1097/MD.0000000000036386, PMID: 38050194 PMC10695622

[ref10] Alkhalaf M. Suwyadi A. AlShamakhi E. Oribi H. Theyab Z. Sumayli I.. (2021). Determinants and prevalence of tobacco smoking among medical students at Jazan University, Saudi Arabia. J. Smok. Cessat. 2021:6632379. doi: 10.1155/2021/6632379, PMID: 34306226 PMC8279191

[ref11] Alkhateeb S. A. Alkhameesi N. F. Lamfon G. N. Khawandanh S. Z. Kurdi L. K. Faran M. Y.. (2019). Pattern of physical exercise practice among university students in the Kingdom of Saudi Arabia (before beginning and during college): a cross-sectional study. BMC Public Health 19, 1–7. doi: 10.1186/s12889-019-8093-2, PMID: 31864325 PMC6925893

[ref12] Alkhawaldeh A. Al Omari O. Al Aldawi S. Al Hashmi I. Ann Ballad C. Ibrahim A.. (2023). Stress factors, stress levels, and coping mechanisms among university students. Sci. World J. 2023, 1–9. doi: 10.1155/2023/2026971, PMID: 37426577 PMC10325878

[ref13] Al-Momani M. M. (2021). Health-promoting lifestyle and its association with the academic achievements of medical students in Saudi Arabia. Pak. J. Med. Sci. 37, 561–566. doi: 10.12669/pjms.37.2.3417, PMID: 33679950 PMC7931320

[ref14] Almoraie N. M. Alothmani N. M. Alomari W. D. Al-amoudi A. H. (2024). Addressing nutritional issues and eating behaviours among university students: a narrative review. Nutr. Res. Rev. 1-16, 1–16. doi: 10.1017/S0954422424000088, PMID: 38356364

[ref15] Almutairi K. M. Alonazi W. B. Vinluan J. M. Almigbal T. H. Batais M. A. Alodhayani A. A.. (2018). Health promoting lifestyle of university students in Saudi Arabia: a cross-sectional assessment. BMC Public Health 18, 1–10. doi: 10.1186/s12889-018-5999-z, PMID: 30185167 PMC6126031

[ref16] Alothman S. A. Al Baiz A. A. Alzaben A. S. Khan R. Alamri A. F. Omer A. B. (2024). Factors associated with lifestyle behaviors among university students: a cross-sectional study. Healthcare 12:154. doi: 10.3390/healthcare12020154 38255042 PMC10815065

[ref17] Alpar R. (2016). Uygulamalı İstatistik ve Geçerlilik-Güvenilirlik. Detay Yayıncılık. Yenilenmiş 4 Baskı, Ankara. Detay Yayıncılık. 647s.

[ref18] Alqahtani J. S. Aldhahir A. M. Alanazi Z. Alsulami E. Z. Alsulaimani M. A. Alqarni A. A.. (2023). Impact of smoking status and nicotine dependence on academic performance of health sciences students. Subst. Abus. Rehabil. 14, 13–24. doi: 10.2147/SAR.S393062, PMID: 36865699 PMC9970882

[ref19] Alshdifat E. Alkhawaldeh A. Albashtawy M. Mohammad K. Al-Rawashdeh S. Malak M.. (2024). Breakfast skipping and associated factors among Jordanian university students. Iran. J. Nurs. Midwifery Res. 29, 125–132. doi: 10.4103/ijnmr.ijnmr_301_22, PMID: 38333350 PMC10849275

[ref20] Amanvermez Y. Karyotaki E. Cuijpers P. Ciharova M. Bruffaerts R. Kessler R. C.. (2023). Sources of stress among domestic and international students: a cross-sectional study of university students in Amsterdam, the Netherlands. Anxiety Stress Coping 37, 428–445. doi: 10.1080/10615806.2023.2280701, PMID: 38047318

[ref21] Amiri M. Chaman R. Khosravi A. (2019). The relationship between health-promoting lifestyle and its related factors with self-efficacy and well-being of students. Osong. Public Health Res. Perspect. 10, 221–227. doi: 10.24171/j.phrp.2019.10.4.04, PMID: 31497493 PMC6711714

[ref22] Andrade M. B. T. Felipe A. O. B. Vedana K. G. G. Scorsolini-Comin F. (2020). The nexus between religiosity/spirituality and suicidal behavior in young people. SMAD, Rev Eletrônica Saúde Mental Álcool Drog 16, 109–121. doi: 10.11606/issn.1806-6976.smad.2020.169257

[ref23] Assaf I. Brieteh F. Tfaily M. El-Baida M. Kadry S. Balusamy B. (2019). Students university healthy lifestyle practice: quantitative analysis. Health İnf. Sci. Syst. 7, 7–12. doi: 10.1007/s13755-019-0068-2, PMID: 30956789 PMC6424984

[ref24] Azmi J. Munirah S. Nurumal M. S. Hani H. (2021). Religiosity and its relationship with smoking cessation: a systematic review. IIUM Med. J. Malays. 20, 85–94. doi: 10.31436/imjm.v20i4

[ref25] Baalmann T. Brömmelhaus A. Hülsemann J. Feldhaus M. Speck K. (2024). The impact of parents, intimate relationships, and friends on students’ dropout intentions. J. Coll. Stud. Retent. 26, 923–947. doi: 10.1177/15210251221133374

[ref26] Badnava S. Moshki M. Pakdaman M. Sahebdel H. (2024). Investigating the conceptualization of spiritual health literacy in university students: an exploratory qualitative study. J. Res. Health 14, 367–374. doi: 10.32598/JRH.14.4.2391.1

[ref27] Bae S. M. (2020). Long term effect of adverse childhood experiences, school disengagement, and reasons for leaving school on delinquency in adolescents who dropout. Front. Psychol. 11:2096. doi: 10.3389/fpsyg.2020.02096, PMID: 33381060 PMC7768077

[ref28] Bafunno D. Catino A. Lamorgese V. Del Bene G. Longo V. Montrone M.. (2020). Impact of tobacco control interventions on smoking initiation, cessation, and prevalence: a systematic review. J. Thorac. Dis. 12, 3844–3856. doi: 10.21037/jtd.2020.02.23, PMID: 32802466 PMC7399441

[ref29] Bahar Z. Beşer A. Gördes N. Ersin F. Kıssal A. (2008). Healthy life style behavior scale II: a reliability and validity study. J. Cumhuriyet Univ. Sch. Nurs. 12, 1–13.

[ref30] Bala S. Babu S. Muralidharan S. Moses M. Hashilkar N. Bhimalli S. (2024). Comparison of traditional lecture and interactive teaching methods in large group teaching of non communicable diseases: a quasi experimental study. Healthline 15, 107–112. doi: 10.51957/Healthline_624_2024

[ref31] Barrington-Trimis J. L. Braymiller J. L. Unger J. B. McConnell R. Stokes A. Leventhal A. M.. (2020). Trends in the age of cigarette smoking initiation among young adults in the US from 2002 to 2018. JAMA Netw. Open 3:e2019022. doi: 10.1001/jamanetworkopen.2020.19022, PMID: 33021650 PMC7539122

[ref32] Baykal D. Kutlu L. Demir B. D. (2022). The correlation between nursing students' healthy lifestyle behaviors, cardiovascular disease risk factors' knowledge level, and obsession symptoms. J. Educ. Health Promot. 11:281. doi: 10.4103/jehp.jehp_902_21, PMID: 36325215 PMC9621382

[ref33] Bayomy H. E. Alruwaili S. M. Alsayer R. I. Alanazi N. K. Albalawi D. A. Al Shammari K. H.. (2024). Eating habits of students of health colleges and non-health colleges at the northern border University in the Kingdom of Saudi Arabia. PLoS One 19:e0312750. doi: 10.1371/journal.pone.0312750, PMID: 39466744 PMC11516010

[ref34] Belfrage A. S. V. Grotmo K. S. Tyssen R. Moum T. Finset A. Rø K. I.. (2018). Factors influencing doctors’ counselling on patients’ lifestyle habits: a cohort study. BJGP Open 2, 1–12. doi: 10.3399/bjgpopen18X101607, PMID: 30564740 PMC6202006

[ref35] Benaich S. Mehdad S. Andaloussi Z. Boutayeb S. Alamy M. Aguenaou H.. (2021). Weight status, dietary habits, physical activity, screen time and sleep duration among university students. Nutr. Health 27, 69–78. doi: 10.1177/0260106020960863 33045923

[ref36] Berardelli I. Corigliano V. Hawkins M. Comparelli A. Erbuto D. Pompili M. (2018). Lifestyle interventions and prevention of suicide. Front. Psych. 9:567. doi: 10.3389/fpsyt.2018.00567, PMID: 30459660 PMC6232529

[ref37] Berg E. Wilhelm K. Handley T. (2020). Should we increase the focus on diet when considering associations between lifestyle habits and deliberate self-harm? BMC Psychiatry 20, 1–8. doi: 10.1186/s12888-020-02950-0, PMID: 33238947 PMC7687696

[ref38] Bernardo A. B. Tuero E. Cervero A. Dobarro A. Galve-González C. (2020). Bullying and cyberbullying: variables that ınfluence university dropout. Comunicar 28, 63–72. doi: 10.3916/C64-2020-06

[ref39] Bożek A. Nowak P. F. Blukacz M. (2020). The relationship between spirituality, health-related behavior, and psychological well-being. Front. Psychol. 11:1997. doi: 10.3389/fpsyg.2020.01997, PMID: 32922340 PMC7457021

[ref40] Brett C. E. Mathieson M. L. Rowley A. M. (2023). Determinants of wellbeing in university students: the role of residential status, stress, loneliness, resilience, and sense of coherence. Curr. Psychol. 42, 19699–19708. doi: 10.1007/s12144-022-03125-8

[ref41] Brown C. E. B. Richardson K. Halil-Pizzirani B. Atkins L. Yücel M. Segrave S. A. (2024). Key influences on university students’ physical activity: a systematic review using the theoretical domains framework and the COM-B model of human behaviour. BMC Public Health 24:418. doi: 10.1186/s12889-023-17621-4, PMID: 38336748 PMC10854129

[ref42] Canino G. J. Shrout P. E. Wall M. Alegria M. Duarte C. S. Bird H. R. (2022). Outcomes of serious antisocial behavior from childhood to early adulthood in two Puerto Rican samples in two contexts. Soc. Psychiatry Psychiatr. Epidemiol. 57, 267–277. doi: 10.1007/s00127-021-02148-z 34357404 PMC9923882

[ref43] Cao J. Wang K. Shi Y. Pan Y. Lyu M. Ji Y.. (2023). Effects of personal and interpersonal factors on changes of food choices and physical activity among college students. PLoS One 18:e0288489. doi: 10.1371/journal.pone.0288489, PMID: 37440487 PMC10343029

[ref44] Casimiro-Andújar A. J. Artés-Rodríguez E. Díez-Fernández D. M. Lirola M. J. (2023). Effects of a physical exercise programme through service-learning methodology on physical activity, physical fitness and perception of physical fitness and health in university students from Spain: a preliminary study. Int. J. Environ. Res. Public Health 20:3377. doi: 10.3390/ijerph20043377, PMID: 36834072 PMC9962317

[ref45] Cass A. L. Holt E. W. Criss S. Hunt E. Reed R. (2021). Health-related priorities, perceptions, and values of university students: implications for wellness education. Am. J. Health Educ. 52, 37–47. doi: 10.1080/19325037.2020.1844103

[ref46] Castro-Cuesta J. Y. Montoro-García S. Sánchez-Macarro M. Carmona Martínez M. Espinoza Marenco I. C. Pérez-Camacho A.. (2023). Adherence to the Mediterranean diet in first-year university students and its association with lifestyle-related factors: a cross-sectional study. Hipertens Riesgo Vasc. 40, 65–74. doi: 10.1016/j.hipert.2022.09.001 36244967

[ref47] Cavus K. Yıldız R. Berent M. (2020). Investigation of first and emergency aid students' status of coping with distress according to physical activity levels. J. Paramed. Emerg. Health Serv. 1, 56–64.

[ref48] Centers for Disease Control and Prevention (CDC). Smoking & tobacco use. Health Effects of Cigarette Smoking. (2021). Available at: https://www.cdc.gov/tobacco/basic_information/health_effects/index.htm (accessed April 13, 2022).

[ref49] Cimene F. T. A. Albino A. A. C. Mijares R. A. J. Hallazgo F. P. M. Austria M. J. Corporal L. M. C.. (2023). Understanding the complex factors behind students dropping out of school. Eur. J. Sci. Innov. Technol. 3, 114–125.

[ref50] Coban A. Adana F. Taspinar A. Arslantas H. (2017). Measuring the effectiveness of healthy life style behaviors course given to university students. Balikesir Health Sci. J. 6, 16–21. doi: 10.5505/bsbd.2017.88597

[ref51] Crispim M. O. Santos C. M. R. D. Frazão I. D. S. Frazão C. M. F. Q. Albuquerque R. C. R. Perrelli J. G. A. (2021). Prevalence of suicidal behavior in young university students: a systematic review with meta-analysis. Rev. Lat. Am. Enfermagem 29:e3495. doi: 10.1590/1518-8345.5320.3495, PMID: 34755776 PMC8584877

[ref52] Dai X. Gakidou E. Lopez A. D. (2022). Evolution of the global smoking epidemic over the past half century: strengthening the evidence base for policy action. Tob. Control. 31, 129–137. doi: 10.1136/tobaccocontrol-2021-056535, PMID: 35241576

[ref53] Dasagi M. Mantey D. S. Harrell M. B. Wilkinson A. V. (2021). Self-reported history of intensity of smoking is associated with risk factors for suicide among high school students. PLoS One 16:e0251099. doi: 10.1371/journal.pone.0251099, PMID: 33983989 PMC8118454

[ref54] Davletbaeva Z. K. Kuvayeva M. M. Musin S. R. (2021). Prevention of antisocial behavior of students. In Bataev D. K. Gapurov S. A. Osmaev A. D. Akaev V. K. Idigova L. M. Ovhadov M. R.. (Eds.), Knowledge, Man and Civilization – ISCKMC 2020, vol 107. European Proceedings of Social and Behavioural Sciences (pp. 337–347). Russia: European Publisher. doi: 10.15405/epsbs.2021.05.46

[ref55] de Vocht F. Katikireddi S. V. McQuire C. Tilling K. Hickman M. Craig P. (2021). Conceptualising natural and quasi experiments in public health. BMC Med. Res. Methodol. 21, 32–38. doi: 10.1186/s12874-021-01224-x, PMID: 33573595 PMC7879679

[ref56] de-Mateo-Silleras B. Camina-Martín M. A. Cartujo-Redondo A. Carreño-Enciso L. de-la-Cruz-Marcos S. Redondo-del-Río P. (2019). Health perception according to the lifestyle of university students. J. Community Health 44, 74–80. doi: 10.1007/s10900-018-0555-4 30014181

[ref57] Dennison C. R. (2022). Dropping out of college and dropping into crime. Justice Q. 39, 585–611. doi: 10.1080/07418825.2020.1740296

[ref58] Desai R. Mercken L. A. Ruiter R. A. Schepers J. Reddy P. S. (2019). Cigarette smoking and reasons for leaving school among school dropouts in South Africa. BMC Public Health 19, 130–110. doi: 10.1186/s12889-019-6454-5, PMID: 30700276 PMC6354377

[ref59] Dewi M. C. S. Pratiwi D. Yulia Y. (2024). Engaging students through powerpoint: the power of multimodal learning. Voices English Lang. Educ. Soc. 8, 337–347. doi: 10.29408/veles.v8i2.24289

[ref60] Diamond J. J. Becker J. A. Arenson C. A. Chambers C. V. Rosenthal M. P. (2007). Development of a scale to measure adults' perceptions of health: preliminary findings. J. Community Psychol. 35, 557–561. doi: 10.1002/jcop.20164

[ref61] Diaz-Serrano L. Stoyanova A. P. (2023). The relationship between overweight and education revisited: a test of the selection hypothesis based on adolescents’ educational aspirations. Public Health 225, 237–243. doi: 10.1016/j.puhe.2023.09.013, PMID: 37944279

[ref62] Dinis M. A. P. Sousa H. F. P. Moura A. D. Viterbo L. M. Pinto R. J. (2019). Health behaviors as a mediator of the association between interpersonal relationships and physical health in a workplace context. Int. J. Environ. Res. Public Health 16:2392. doi: 10.3390/ijerph16132392, PMID: 31284473 PMC6651570

[ref63] Dmitruk A. Hołub W. (2024). Physical activity and other forms of spending free time by first-year nursing students depending on their age. Rozprawy Społeczne 18, 398–414. doi: 10.29316/rs/190734

[ref64] Du C. Luo N. Wu L. Gadd S. Zhang X. Tucker R. M. (2023). Health behavior changes associated with weight gain among first-year international students studying at an American university. J. Am. Coll. Heal. 71, 300–309. doi: 10.1080/07448481.2021.1891082 33651676

[ref65] Edelmann D. Pfirrmann D. Heller S. Dietz P. Reichel J. L. Werner A. M.. (2022). Physical activity and sedentary behavior in university students–the role of gender, age, field of study, targeted degree, and study semester. Front. Public Health 10:821703. doi: 10.3389/fpubh.2022.821703, PMID: 35784227 PMC9244168

[ref66] Egger G. Binns A. Rossner S. Sagner M. (2017). Lifestyle medicine: Lifestyle, the environment and preventive medicine in health and disease. Cambridge, MA: Academic Press.

[ref67] Engin E. Cuhadar D. Ozturk E. (2012). Healthy life behaviors and suicide probability in university students. Arch. Psychiatr. Nurs. 26, 43–53. doi: 10.1016/j.apnu.2011.05.001 22284079

[ref68] Erenoglu R. Can R. Sekerci Y. G. (2019). The effect of the health promotion program for young people on health behaviors, health perception, and self-efficacy levels: a randomized controlled trial. Int. J. Caring Sci. 12:1203.

[ref69] Eymirli S. P. Mustuloglu S. Koksal E. Turgut M. D. Tekcicek M. U. (2024). Dental students’ healthy lifestyle behaviors, physical activity levels and social media use: cross sectional study. Discov. Public Health 21:95. doi: 10.1186/s12982-024-00215-9

[ref70] Fashafsheh I. Al-Ghabeesh S. H. Ayed A. Salama B. Batran A. Bawadi H. (2021). Health promoting behaviors among nursing students: Palestinian perspective. Inquiry 58:00469580211018790. doi: 10.1177/00469580211018790, PMID: 34014137 PMC8141992

[ref71] Feizi S. Nasiri M. Bahador H. Amiri M. H. Mirhosseini H. (2020). The relationship between spiritual well-being and happiness among healthcare students: application of the spiritual health questionnaire for the Iranian population. Heliyon 6:e05448. doi: 10.1016/j.heliyon.2020.e05448, PMID: 33241140 PMC7672288

[ref72] Fernández-Suárez A. Herrero J. Pérez B. Juarros-Basterretxea J. Rodríguez-Díaz F. J. (2016). Risk factors for school dropout in a sample of juvenile offenders. Front. Psychol. 7:1993. doi: 10.3389/fpsyg.2016.01993, PMID: 28082934 PMC5183606

[ref73] Frías Armenta M. Corral-Frías N. S. (2021). Positive university environment and agreeableness as protective factors against antisocial behavior in Mexican university students. Front. Psychol. 12:662146. doi: 10.3389/fpsyg.2021.662146, PMID: 34366980 PMC8339411

[ref74] Friedenreich C. M. Ryder-Burbidge C. McNeil J. (2021). Physical activity, obesity and sedentary behavior in cancer etiology: epidemiologic evidence and biologic mechanisms. Mol. Oncol. 15, 790–800. doi: 10.1002/1878-0261.12772 32741068 PMC7931121

[ref75] Ganson K. T. O'Connor J. Nagata J. M. (2022). Physical violence perpetration among college students: prevalence and associations with substance use and mental health symptoms. J. Interpers. Violence 37, NP11110–NP11134. doi: 10.1177/0886260521991888, PMID: 33535868

[ref76] Genctanırım D. (2014). University form of risk behavaiors scale: Vadility and reability studies. J. Meas. Eval. Educ. Psychol. 5, 24–34.

[ref77] Ghazawy E. R. Mahfouz E. M. Rahman A. E. Ahmed T. Emam S. A. (2022). Obesity/overweight among university students. Minia, Egypt. Minia J. Med. Res. 33, 30–36. doi: 10.21608/mjmr.2022.129100.1068

[ref78] Ghorabi S. T. Jalilian M. Sadeghifar J. Zavareh M. S. A. (2021). Investigation of health-promoting behaviors of employees of medical university: a perspective from west of Iran. J. Educ. Health Promot. 10:139. doi: 10.4103/jehp.jehp_835_20, PMID: 34222514 PMC8224491

[ref79] Gilan A. B. Janatolmakan M. Ashtarian H. Rezaei M. Khatony A. (2021). Health promoting lifestyle and associated factors among medical sciences students in Kermanshah, Iran: a cross-sectional study. J. Environ. Public Health 2021:6691593. doi: 10.1155/2021/6691593, PMID: 33995535 PMC8096551

[ref80] Grant J. E. Odlaug B. L. Lust K. Christenson G. (2016). Characteristics and correlates of stealing in college students. Crim. Behav. Ment. Health 26, 101–109. doi: 10.1002/cbm.1986 26648022

[ref81] Gubbels J. van der Put C. E. Assink M. (2019). Risk factors for school absenteeism and dropout: a meta-analytic review. J. Youth Adolesc. 48, 1637–1667. doi: 10.1007/s10964-019-01072-5, PMID: 31312979 PMC6732159

[ref82] Guedes D. P. de Lima K. A. dos Santos Silva A. L. (2024). Prevalence and correlates of health risk behaviors among university students from a state in the southern region of Brazil. Int. J. Environ. Res. Public Health 21:612. doi: 10.3390/ijerph21050612, PMID: 38791826 PMC11120948

[ref83] Gulnar E. Asık E. Ozveren H. (2024). The effect of a stress management program on first-year nursing students’ clinical stress: a randomized controlled experimental study. Nurse Educ. Today 136:106131. doi: 10.1016/j.nedt.2024.106131, PMID: 38368735

[ref84] Haase E. Schönfelder A. Nesterko Y. Glaesmer H. (2022). Prevalence of suicidal ideation and suicide attempts among refugees: a meta-analysis. BMC Public Health 22, 1–12. doi: 10.1186/s12889-022-13029-8, PMID: 35365108 PMC8976302

[ref85] Habbash A. S. Alshabab M. Q. A. Alqahtani F. M. R. Alhussain R. M. O. Alshehri N. A. Benjamin L. S.. (2023). Prevalence of smoking among university students, Aseer region in Saudi Arabia – a cross-sectional study. J. Popul. Ther. Clin. Pharmacol. 30, 1–10. doi: 10.47750/jptcp.2023.30.13.001

[ref86] Haider N. Abbas U. Arif H. E. Uqaili A. A. Khowaja M. A. Hussain N.. (2024). From plate to profile: investigating the influence of dietary habits and inactive lifestyle on lipid profile in medical students at clerkship. BMC Nutr. 10:71. doi: 10.1186/s40795-024-00871-9, PMID: 38715144 PMC11077723

[ref87] Hassan M. S. Hossain M. K. Khan H. T. A. (2019). Prevalence and predictors of tobacco smoking among university students in Sylhet division, Bangladesh. Int. Health 11, 306–313. doi: 10.1093/inthealth/ihy091, PMID: 30517660

[ref88] Heradstveit O. Hysing M. Bøe T. Nilsen S. A. Sivertsen B. Bretteville-Jensen A. L.. (2024). Prospective associations between adolescent risky substance use and school dropout and the role of externalising and internalising problems. Nordic Stud. Alcohol Drugs 41, 24–38. doi: 10.1177/14550725231188568, PMID: 38356785 PMC10863553

[ref89] Hjarnaa L. Møller S. P. Curtis A. B. Becker U. Andersen O. Torvik F. A.. (2023). Alcohol intake and academic performance and dropout in high school: a prospective cohort study in 65,233 adolescents. J. Adolesc. Health 73, 1083–1092. doi: 10.1016/j.jadohealth.2023.07.008 37702649

[ref90] Hsu J. L. Goldsmith G. R. (2021). Instructor strategies to alleviate stress and anxiety among college and university STEM students. CBE Life Sci. Educ. 20:es1. doi: 10.1187/cbe.20-08-0189, PMID: 33635124 PMC8108494

[ref91] Htet H. Saw Y. M. Saw T. N. Htun N. M. M. Lay Mon K. Cho S. M.. (2020). Prevalence of alcohol consumption and its risk factors among university students: a cross-sectional study across six universities in Myanmar. PLoS One 15:e0229329. doi: 10.1371/journal.pone.0229329 32084226 PMC7034886

[ref92] Hutchesson M. J. Whatnall M. C. Yazin N. Fenton S. Duncan M. J. Kay-Lambkin F. J.. (2022). Health behavior interventions for university students measuring mental health outcomes: a scoping review. Front. Public Health 10:1063429. doi: 10.3389/fpubh.2022.1063429, PMID: 36568797 PMC9771454

[ref93] Hwang Y. Oh J. (2020). Factors affecting health-promoting behaviors among nursing students. Int. J. Environ. Res. Public Health 17:6291. doi: 10.3390/ijerph17176291, PMID: 32872367 PMC7503930

[ref94] Ikoona E. N. Toure M. A. Njenga A. Namulemo L. Kaluya R. Kamara K.. (2023). Prevalence and factors associated with underweight among 15-49-year-old women in Sierra Leone: a secondary data analysis of Sierra Leone demographic health survey of 2019. BMC Womens Health 23:192. doi: 10.1186/s12905-023-02358-4, PMID: 37085835 PMC10122406

[ref95] Ilić M. Pang H. Vlaški T. G. Grujičić M. Novaković B. (2024). Prevalence and associated factors of overweight and obesity among medical students from the Western Balkans (south-East Europe region). BMC Public Health 24:29. doi: 10.1186/s12889-023-17389-7, PMID: 38166959 PMC10763029

[ref96] Intiful F. D. Steele-Dadzie R. Amos P. M. Pobee R. Ainuson-Quampah J. Ammah C.. (2021). “The impact of interpersonal relationships on dietary habits” in Interpersonal relationships. IntechOpen ed. Martha Peaslee Levine. doi: 10.5772/intechopen.95482

[ref97] Irfan M. Jabbar M. Hameed S. (2019). Dietary habits and prevalence of underweight/obesity in students of University of Gujrat, Pakistan. J. Liaquat Univ. Med. Health Sci. 18, 175–180. doi: 10.22442/jlumhs.191820623

[ref98] Isaac V. Wu C. Y. McLachlan C. S. Lee M. B. (2018). Associations between health-related self-efficacy and suicidality. BMC Psychiatry 18, 126–128. doi: 10.1186/s12888-018-1705-z, PMID: 29747578 PMC5946427

[ref99] Islam S. Akter R. Sikder T. Griffiths M. D. (2022). Prevalence and factors associated with depression and anxiety among first-year university students in Bangladesh: a cross-sectional study. Int. J. Ment. Heal. Addict. 20, 1289–1302. doi: 10.1007/s11469-020-00242-y

[ref100] Jeffrey N. K. Senn C. Y. Krieger M. A. Forrest A. (2022). The scope, nature, and impact of sexual violence among students from a Canadian university: a random sample study. Can. J. Behav. Sci. 55, 100–112. doi: 10.1037/cbs0000329

[ref101] JohH. K.KwonH.SonK. Y.YunJ. M.ChoS. H.HanK.. (2024). Trends in underweight and severe underweight disparities in Korean adults and older adults: a nationwide, repeated cross-sectional study. J. Nutr. Health Aging 28:100185. doi: 10.1016/j.jnha.2024.10018538341966

[ref102] Kabbash I. A. Salama B. Mohammad M. A. Galal H. Yousef N. Elghazally N. M. (2023). Perception and experiences of suicide among university students in Egypt. Middle East Curr. Psychiatry 30:87. doi: 10.1186/s43045-023-00358-6

[ref103] Kabir R. Syed H. Z. Vinnakota D. Okello S. Isigi S. S. Abdul Kareem S. K.. (2024). Suicidal behaviour among the university students in the UK: a systematic review. Heliyon 10:e24069. doi: 10.1016/j.heliyon.2024.e24069, PMID: 38293523 PMC10826643

[ref104] Kadıoğlu H. Yıldız A. (2012). Validity and reliability of turkish version of perception of health scale. J. Med. Sci. 32, 47–53. doi: 10.5336/medsci.2010-21761

[ref105] Kaggwa M. M. Arinaitwe I. Muwanguzi M. Nduhuura E. Kajjimu J. Kule M.. (2022). Suicidal behaviours among Ugandan university students: a cross-sectional study. BMC Psychiatry 22, 1–13. doi: 10.1186/s12888-022-03858-7, PMID: 35365105 PMC8972906

[ref106] Kallio J. Hakonen H. Syväoja H. Kulmala J. Kankaanpää A. Ekelund U.. (2020). Changes in physical activity and sedentary time during adolescence: gender differences during weekdays and weekend days. Scand. J. Med. Sci. Sports 30, 1265–1275. doi: 10.1111/sms.13668, PMID: 32248577 PMC7318293

[ref107] Kamruzzaman M. Hossain A. Islam M. A. Ahmed M. S. Kabir E. Khan M. N. (2024). Exploring the prevalence of depression, anxiety, and stress among university students in Bangladesh and their determinants. Clin. Epidemiol. Glob. Health 28:101677. doi: 10.1016/j.cegh.2024.101677

[ref108] Karletsos D. Hutchinson P. Leyton A. Meekers D. (2021). The effect of interpersonal communication in tobacco control campaigns: a longitudinal mediation analysis of a Ghanaian adolescent population. Prev. Med. 142:106373. doi: 10.1016/j.ypmed.2020.106373, PMID: 33340636

[ref109] Karyotaki E. Cuijpers P. Albor Y. Alonso J. Auerbach R. P. Bantjes J.. (2020). Sources of stress and their associations with mental disorders among college students: results of the world health organization world mental health surveys international college student initiative. Front. Psychol. 11:1759. doi: 10.3389/fpsyg.2020.01759 32849042 PMC7406671

[ref110] Katzmarzyk P. T. Friedenreich C. Shiroma E. J. Lee I. M. (2022). Physical inactivity and non-communicable disease burden in low-income, middle-income and high-income countries. Br. J. Sports Med. 56, 101–106. doi: 10.1136/bjsports-2020-103640, PMID: 33782046 PMC8478970

[ref111] Khademian F. Aslani A. Ravangard R. Nami M. Abbasi S. Bastani P. (2021). Iranian university students’ stressors and coping strategies: a qualitative study. J. Educ. Health Promot. 10:244. doi: 10.4103/jehp.jehp_1278_20, PMID: 34485541 PMC8395881

[ref112] Khurram F. B. Hossain M. O. Korobi F. T. J. Khan M. Talukder M. U. Nath C. D.. (2023). Understanding school dropout and its impact on the community in the Khulna region of Bangladesh. Open J. Soc. Sci. 11, 582–596. doi: 10.4236/jss.2023.1111038

[ref113] Kim H. Ryu S. Jeon H. J. Roh S. (2023). Lifestyle factors and suicide risk: a nationwide population-based study. J. Affect. Disord. 328, 215–221. doi: 10.1016/j.jad.2023.02.044, PMID: 36806600

[ref114] Kim J. S. Seo Y. (2023). Breakfast habits, sedentary behavior, and suicide among Korean adolescents: a cross-sectional national study. PLoS One 18:e0285312. doi: 10.1371/journal.pone.0285312, PMID: 37141205 PMC10159120

[ref115] Kim K. B. Shin Y. A. (2020). Males with obesity and overweight. J. Obes. Metab. Syndr. 29, 18–25. doi: 10.7570/jomes20008, PMID: 32146733 PMC7117999

[ref116] Kim C. H. Song Y. E. Jeon Y. J. (2021). The effect of college students’ physical activity level on depression and personal relationships. Healthcare 9:526. doi: 10.3390/healthcare9050526, PMID: 33947125 PMC8145648

[ref117] Lansford J. E. Dodge K. A. Pettit G. S. Bates J. E. (2016). A public health perspective on school dropout and adult outcomes: a prospective study of risk and protective factors from age 5 to 27 years. J. Adolesc. Health 58, 652–658. doi: 10.1016/j.jadohealth.2016.01.014, PMID: 27009741 PMC4877222

[ref118] Lanza H. I. Huang D. Y. (2015). Is obesity associated with school dropout? Key developmental and ethnic differences. J. Sch. Health 85, 663–670. doi: 10.1111/josh.12295, PMID: 26331748 PMC4603989

[ref119] Lee S. M. Jeong H. C. So W. Y. Youn H. S. (2020). Mediating effect of sports participation on the relationship between health perceptions and health promoting behavior in adolescents. Int. J. Environ. Res. Public Health 17:6744. doi: 10.3390/ijerph17186744, PMID: 32947940 PMC7559390

[ref120] Leung C. H. Pong H. K. (2021). Cross-sectional study of the relationship between the spiritual wellbeing and psychological health among university students. PLoS One 16:e0249702. doi: 10.1371/journal.pone.0249702 33857211 PMC8049307

[ref121] Li X. Chi G. Taylor A. Chen S. T. Memon A. R. Zhang Y.. (2021). Lifestyle behaviors and suicide-related behaviors in adolescents: cross-sectional study using the 2019 YRBS data. Front. Public Health 9:766972. doi: 10.3389/fpubh.2021.766972, PMID: 34926387 PMC8678606

[ref122] Lian Z. Wallace B. C. (2020). Prevalence of past-year mental disorders and its correlates among Chinese international students in US higher education. J. Am. Coll. Heal. 68, 176–184. doi: 10.1080/07448481.2018.1538147, PMID: 30485158

[ref123] Liu C. Sun Z. (2023). The relationship between physical activity and interpersonal distress in college students: the chain mediating role of self-control and mobile phone addiction. Psicologia 36:18. doi: 10.1186/s41155-023-00261-3, PMID: 37530865 PMC10397154

[ref124] Liu W. Wang L. Jiang R. Wang L. Zhang W. Dai F.. (2019). Effects of a healthy lifestyle and behavior-related knowledge intervention on college students in Huai’an City, Jiangsu Province. J. Public Health Emerg. 3:18. doi: 10.21037/jphe.2019.12.03

[ref125] López-Moreno M. Garcés-Rimón M. Miguel-Castro M. Fernández-Martínez E. Iglesias López M. T. (2023). Effect of nutrition education on health science university students to improve cardiometabolic profile and inflammatory status. Nutrients 15:4685. doi: 10.3390/nu15214685, PMID: 37960339 PMC10648054

[ref126] Luszczynska A. Haynes C. (2009). Changing nutrition, physical activity and body weight among student nurses and midwives: effects of a planning intervention and self-efficacy beliefs. J. Health Psychol. 14, 1075–1084. doi: 10.1177/1359105309342290, PMID: 19858328

[ref127] Maazallahi M. Ghonchepour A. Sohrabi M. Golestani Z. Parandeh Afshar P. Malakoutikhah A.. (2021). Spiritual well-being among medical and nonmedical science students. Scientifica 2021:6614961. doi: 10.1155/2021/6614961, PMID: 33986969 PMC8093076

[ref128] Mahfouz A. A. Alsaleem S. A. Alsaleem M. A. Ghazy R. M. (2024). Prevalence of obesity and associated dietary habits among medical students at King Khalid University, Southwestern Saudi Arabia. Medicina 60:347. doi: 10.3390/medicina60030347, PMID: 38541073 PMC10972436

[ref129] Makkawy E. Alrakha A. M. Al-Mubarak A. F. Alotaibi H. T. Alotaibi N. T. Alasmari A. A.. (2021). Prevalence of overweight and obesity and their associated factors among health sciences college students, Saudi Arabia. J. Family Med. Prim. Care 10, 961–967. doi: 10.4103/jfmpc.jfmpc_1749_20, PMID: 34041105 PMC8138400

[ref130] Mamun M. A. Rayhan I. Akter K. Griffiths M. D. (2020). Prevalence and predisposing factors of suicidal ideation among the university students in Bangladesh: a single-site survey. Int. J. Ment. Heal. Addict. 20, 1958–1971. doi: 10.1007/s11469-020-00403-z

[ref131] Manchester R. (2020). How has the health of college students changed in the last 50 years? J. Am. Coll. Heal. 68, 795–797. doi: 10.1080/07448481.2020.1859853 33571080

[ref132] Marendić M. Aranza D. Aranza I. Vladislavić S. Kolčić I. (2024). Differences between health and non-health science students in lifestyle habits, perceived stress and psychological well-being: a cross-sectional study. Nutrients 16:620. doi: 10.3390/nu16050620, PMID: 38474748 PMC10935260

[ref133] Martínez-Otero V. Gaeta M. L. (2022). Educational prevention of antisocial and delinquent behavior in Brazilian adolescents. Psicothema 34, 544–552. doi: 10.7334/psicothema2022.118, PMID: 36268959

[ref134] Masini A. Salussolia A. Anastasia A. G.-C. Grao-Cruces A. Soldà G. Zanutto G.. (2024). Evaluation of school-based interventions including homework to promote healthy lifestyles: a systematic review with meta-analysis. J. Public Health. doi: 10.1007/s10389-024-02239-6

[ref135] Mathad M. D. Rajesh S. K. Pradhan B. (2019). Spiritual well-being and its relationship with mindfulness, self-compassion and satisfaction with life in baccalaureate nursing students: a correlation study. J. Relig. Health 58, 554–565. doi: 10.1007/s10943-017-0532-8 29214405

[ref136] Mayo X. Luque-Casado A. Jimenez A. del Villar F. (2020). Physical activity levels for girls and young adult women versus boys and young adult men in Spain: a gender gap analysis. Sustain. For. 12:6265. doi: 10.3390/su12156265

[ref137] McCarthy C. Warne J. P. (2022). Gender differences in physical activity status and knowledge of Irish university staff and students. Sport Sci. Health 18, 1283–1291. doi: 10.1007/s11332-022-00898-0

[ref138] McCloskey M. S. Ammerman B. A. (2018). Suicidal behavior and aggression-related disorders. Curr. Opin. Psychol. 22, 54–58. doi: 10.1016/j.copsyc.2017.08.010, PMID: 28829989

[ref139] McCoy D. Gill N. Taylor V. C. Thompson D. Bell S. (2020). Examining the relationship between cigarette usage and the influence of interpersonal relationships. J. Stud. Res. doi: 10.47611/jsr.vi.825

[ref140] Michael S. L. Lowry R. Merlo C. Cooper A. C. Hyde E. T. McKeon R. (2020). Physical activity, sedentary, and dietary behaviors associated with indicators of mental health and suicide risk. Prev. Med. Rep. 19:101153. doi: 10.1016/j.pmedr.2020.101153, PMID: 32670781 PMC7350137

[ref141] Mkid S. Strd T. Bmhsk B. (2021). A survey to evaluate the examination stress of physiotherapy undergraduates of faculty of allied health sciences, university of Peradeniya, Sri Lanka. Int. J. Trend Sci. Res. Dev. 5, 2319–7064.

[ref142] Montes G. C. Mendes L. (2021). Effects of violence on school dropout: a panel data analysis to Rio de Janeiro. J. Dev. Areas 55, 329–354. doi: 10.1353/jda.2021.0093

[ref143] Murofushi Y. Yamaguchi S. Kadoya H. Otsuka H. Ogura K. Kaga H.. (2023). Multidimensional background examination of young underweight Japanese women: focusing on their dieting experiences. Front. Public Health 11:1130252. doi: 10.3389/fpubh.2023.1130252, PMID: 37333534 PMC10273403

[ref144] Muscogiuri G. Verde L. Vetrani C. Barrea L. Savastano S. Colao A. (2024). Obesity: Agender-view. J. Endocrinol. Investig. 47, 299–306. doi: 10.1007/s40618-023-02196-z, PMID: 37740888 PMC10859324

[ref145] Musić L. Mašina T. Puhar I. Plančak L. Kostrić V. Kobale M.. (2021). Assessment of health-promoting lifestyle among dental students in Zagreb, Croatia. Dent. J. 9:28. doi: 10.3390/dj9030028, PMID: 33800870 PMC8000712

[ref146] Nagashima Y. Inokuchi M. Sato Y. Hasegawa T. (2024). Underweight in young Japanese women over time: a longitudinal retrospective study of the change in body mass index from ages 6 to 20 years. Ann. Hum. Biol. 51:2345393. doi: 10.1080/03014460.2024.2345393, PMID: 38685666

[ref147] Nakano M. Yamazaki C. Teshirogi H. Kubo H. Ogawa Y. Kameo S.. (2022). How worries about ınterpersonal relationships, academic performance, family support, and classmate social capital ınfluence suicidal ıdeation among adolescents in Japan. Tohoku J. Exp. Med. 256, 73–84. doi: 10.1620/tjem.256.73, PMID: 35082185

[ref148] Nawsherwan Ul Haq I. Tian Q. Ahmed B. Nisar M. Inayat H. Z.. (2021). Assessment of nutrition knowledge among university students: a systematic review. Prog. Nutr. 23:e2021059. doi: 10.23751/pn.v23i2.9374

[ref149] Ndung'u J. Waudo J. Kobia J. (2024). Assessment of nutritional status among undergraduate students at a Nairobi tertiary institution using bmı and waist circumference metrics. Afr. J. Nutr. Diet. 3, 76–89. doi: 10.58460/ajnd.v3i1.66

[ref150] Ndupu L. B. Faghy M. Staples V. Lipka S. Bussell C. (2023). Exploring the predictors of physical inactivity in a university setting. BMC Public Health 23:59. doi: 10.1186/s12889-022-14953-5, PMID: 36624482 PMC9830702

[ref151] Niyogisubizo J. Liao L. Nziyumva E. Murwanashyaka E. Nshimyumukiza P. C. (2022). Predicting student's dropout in university classes using two-layer ensemble machine learning approach: a novel stacked generalization. Comput. Educ. 3:100066. doi: 10.1016/j.caeai.2022.100066

[ref152] O'Connor D. B. Thayer J. F. Vedhara K. (2021). Stress and health: a review of psychobiological processes. Annu. Rev. Psychol. 72, 663–688. doi: 10.1146/annurev-psych-062520-122331, PMID: 32886587

[ref153] Oguz S. Camcı G. Kazan M. (2018). The prevalence of cigarette smoking and knowing status for diseases caused by smoking among students of university. Van Med. J. 25, 332–337. doi: 10.5505/vtd.2018.02411

[ref154] Okwudili A. Chibuike O. Philip O. Ekpunobi C. Okorieh A. Grace O.. (2020). The study of drug use, spirituality, intimacy and age as determinants of antisocial behaviour among youths. Open Access Libr. J. 7, 1–31. doi: 10.4236/oalib.1106431

[ref155] Oral B. Cetinkaya F. (2020). Health perceptions and healthy lifestyle behaviors of Erciyes University students. Med. Sci. 9, 829–836. doi: 10.5455/medscience.2020.05.076

[ref156] Owusu-Ansah F. E. Addae A. A. Peasah B. O. Oppong Asante K. Osafo J. (2020). Suicide among university students: prevalence, risks and protective factors. Health Psychol. Behav. Med. 8, 220–233. doi: 10.1080/21642850.2020.1766978 34040869 PMC8114407

[ref157] Palamutoglu M. I. Kose G. Abacı M. Gok F. R. (2024). The impact of university students' accommodation environments on their dietary choices. Food Health 10, 149–159. doi: 10.3153/FH24014

[ref158] Pascoe M. C. Hetrick S. E. Parker A. G. (2020). The impact of stress on students in secondary school and higher education. Int. J. Adolesc. Youth 25, 104–112. doi: 10.1080/02673843.2019.1596823

[ref159] Pfledderer C. D. Bai Y. Brusseau T. A. Burns R. D. King Jensen J. L. (2022). Changes in college students' health behaviors and substance use after a brief wellness intervention during COVID-19. Prev. Med. Rep. 26:101743. doi: 10.1016/j.pmedr.2022.101743, PMID: 35242504 PMC8885082

[ref160] Phelps N. H. Singleton R. K. Zhou B. Heap R. A. Mishra A. Bennett J. E.. (2024). Worldwide trends in underweight and obesity from 1990 to 2022: a pooled analysis of 3663 population-representative studies with 222 million children, adolescents, and adults. Lancet 403, 1027–1050. doi: 10.1016/S0140-6736(23)02750-2 38432237 PMC7615769

[ref161] Pınar S. E. (2020). Health related risky behaviors of university students studying at midwifery department. Lokman Hekim J. 10, 458–467. doi: 10.31020/mutftd.723914

[ref162] Pitil P. P. Ghazali S. R. (2022). Overweight and obesity: a study among university students in Sarawak, Malaysia. Int. J. Health Promot. Educ. 1-13, 1–13. doi: 10.1080/14635240.2022.2040380

[ref163] Puente-Hidalgo S. Prada-García C. Benítez-Andrades J. A. Fernández-Martínez E. (2024). Promotion of healthy habits in university students: literature review. Healthcare (Basel) 12:993. doi: 10.3390/healthcare12100993, PMID: 38786404 PMC11121720

[ref164] Rabanales-Sotos J. Evangelina Villanueva-Benites M. Jacinto-Magallanes-Castilla J. Leitón-Espinoza Z. E. López-González Á. López-Torres-Hidalg J. (2020). Prevalence of overweight and obesity among health sciences students in the Amazonia region of Peru. Healthcare 8:538. doi: 10.3390/healthcare8040538, PMID: 33291580 PMC7761865

[ref165] Radwan H. Hasan H. A. Ismat H. Hakim H. Khalid H. Al-Fityani L.. (2019). Body mass index perception, body image dissatisfaction and their relations with weight-related behaviors among university students. Int. J. Environ. Res. Public Health 16:1541. doi: 10.3390/ijerph16091541, PMID: 31052368 PMC6539402

[ref166] Ramani S. Pathak A. Dalal V. Paul A. Biswas S. (2020). Oxidative stress in autoimmune diseases: an under dealt malice. Curr. Protein Pept. Sci. 21, 611–621. doi: 10.2174/1389203721666200214111816, PMID: 32056521

[ref167] Ramesh S. Yadav P. Dabhade S. (2021). A exploratory study to assess the food choices among college students of Pune City. Indian J. Forensic Med. Toxicol. 15, 831–840. doi: 10.37506/ijfmt.v15i2.14416

[ref168] Ramón-Arbués E. Granada-López J. M. Martínez-Abadía B. Echániz-Serrano E. Antón-Solanas I. Jerue B. A. (2021). Factors related to diet quality: a cross-sectional study of 1055 university students. Nutrients 13:3512. doi: 10.3390/nu13103512 34684513 PMC8537817

[ref169] Ricci F. Modenese A. Gobba F. Morlini I. (2022). Evaluation of an online course promoting health and wellbeing for university students and employees. Eur. J. Investig. Health Psychol. Educ. 12, 1369–1390. doi: 10.3390/ejihpe12090096, PMID: 36135234 PMC9497908

[ref170] Rotich S. Kamau J. Oketch M. A., and, Okube O. T. (2023). Prevalence and predictors of obesity among undergraduate students at a private university, Nairobi, Kenya. Open J. Endocr. Metab. Dis. 13, 23–38., doi: 10.4236/ojemd.2023.132003

[ref171] Ruiz L. D. Zuelch M. L. Dimitratos S. M. Scherr R. E. (2019). Adolescent obesity: diet quality, psychosocial health, and cardiometabolic risk factors. Nutrients 12:43. doi: 10.3390/nu12010043, PMID: 31877943 PMC7020092

[ref172] Samara A. A. Rachiotis G. Pettemeridou S. Papastamatiou K. Tourlakopoulos K. Chelioti E.. (2020). Prevalence of tobacco use, exposure to secondhand smoke and knowledge on smoking cessation among students of health professions in Central Greece: a cross-sectional study. BMJ Open 10:e036512. doi: 10.1136/bmjopen-2019-036512, PMID: 33087367 PMC7580046

[ref173] Sánchez-Ojeda M. A. Roldán C. Melguizo-Rodríguez L. de Luna-Bertos E. (2022). Analysis of the lifestyle of Spanish undergraduate nursing students and comparison with students of other degrees. Int. J. Environ. Res. Public Health 19:5765. doi: 10.3390/ijerph19095765, PMID: 35565155 PMC9103797

[ref174] Santana E. E. S. Neves L. M. Souza K. C. Mendes T. B. Rossi F. E. Silva A. A. D.. (2023). Physically inactive undergraduate students exhibit more symptoms of anxiety, depression, and poor quality of life than physically active students. Int. J. Environ. Res. Public Health 20:4494. doi: 10.3390/ijerph20054494, PMID: 36901511 PMC10001626

[ref175] Schramlová M. Řasová K. Jonsdottir J. Pavlíková M. Rambousková J. Äijö M.. (2024). Quality of life and quality of education among physiotherapy students in Europe. Front. Med. 11:1344028. doi: 10.3389/fmed.2024.1344028, PMID: 38482532 PMC10936755

[ref176] Senarath M. K. I. D. Thalwaththe S. T. R. D. Tennakoon S. U. B. (2021). Evaluation of the quality of life among undergraduates of Faculty of Allied Health Sciences, University of Peradeniya. Int. J. Sci. Appl. Res. 8, 1–10.

[ref177] Sharareh P. Leili T. Abbas M. Jalal P. Ali G. (2020). Determining correlates of the average number of cigarette smoking among college students using count regression models. Sci. Rep. 10, 8874–8810. doi: 10.1038/s41598-020-65813-4, PMID: 32483160 PMC7264191

[ref178] Sharry M. P. Timmins F. (2016). An evaluation of the effectiveness of a dedicated health and well being course on nursing students' health. Nurse Educ. Today 44, 26–32. doi: 10.1016/j.nedt.2016.05.004, PMID: 27429326

[ref179] Shegute T. Wasihun Y. (2021). Prevalence of substance use in university students, Ethiopia. Subst. Abuse 15:15. doi: 10.1177/11782218211003558, PMID: 33854324 PMC8013928

[ref180] Shekari F. Habibi P. Nadrian H. Mohammadpoorasl A. (2020). Health-risk behaviors among Iranian university students, 2019: a web-based survey. Arch. Public Health 78, 131–136. doi: 10.1186/s13690-020-00514-y, PMID: 33298189 PMC7727248

[ref181] Silva A. F. D. Júnior C. A. S. A. Hinnig P. D. F. Lima L. R. A. D. Silva D. A. S. (2020). Suicidal behaviors and sedentary lifestyles among adolescents: a cross-sectional epidemiological study in Latin American and Caribbean countries. Clinics 75:e2015. doi: 10.6061/clinics/2020/e2015 33146359 PMC7561067

[ref182] Sınan O. Sahin S. Sahin S. Unsal A. (2024). Assessment of the level of personal hygıene knowledge and health perceptıon among unıversıty students. J. Health Scı. 33, 52–59. doi: 10.34108/eujhs.1328944

[ref183] Sinnathamby H. Saupin S. Awang Lukman K. Sidek Ahmad Z. N. Salvaraji L. Robinson F. (2023). Prevalence and associated risk factors related to e-cigarette use among students in universiti Malaysia sabah. Malays. J. Public Health Med. 23, 13–21. Available at: https://mjphm.org/index.php/mjphm/article/view/1820

[ref184] Sivanantham P. Sahoo J. Lakshminarayanan S. Bobby Z. Kar S. S. (2021). Profile of risk factors for non-communicable diseases (NCDs) in a highly urbanized district of India: findings from Puducherry district-wide STEPS survey, 2019–20. PLoS One 16:e0245254. doi: 10.1371/journal.pone.0245254, PMID: 33434194 PMC7802941

[ref185] Solhi M. Azar F. E. F. Abolghasemi J. Maheri M. Irandoost S. F. Khalili S. (2020). The effect of educational intervention on health-promoting lifestyle: intervention mapping approach. J. Educ. Health Promot. 9:196. doi: 10.4103/jehp.jehp_768_19, PMID: 33062729 PMC7530417

[ref186] Sosu E. M. Pheunpha P. (2019). Trajectory of university dropout: investigating the cumulative effect of academic vulnerability and proximity to family support. Front. Educ. 4:6. doi: 10.3389/feduc.2019.00006

[ref187] Souto T. S. Ramires A. Leite Â. Santos V. Santo R. E. (2018). Health perception: validation of a scale for the portuguese population. Trends Psychol. 26, 2167–2183. doi: 10.9788/TP2018.4-17Pt

[ref188] Svansdottir E. Arngrimsson S. A. Sveinsson T. Johannsson E. (2015). Importance of physical health and health-behaviors in adolescence for risk of dropout from secondary education in young adulthood: an 8-year prospective study. Int. J. Equity Health 14, 1–11. doi: 10.1186/s12939-015-0272-x, PMID: 26597711 PMC4657320

[ref189] Tabrizi J. S. Doshmangir L. Khoshmaram N. Shakibazadeh E. Abdolahi H. M. Khabiri R. (2024). Key factors affecting health promoting behaviors among adolescents: a scoping review. BMC Health Serv. Res. 24:58. doi: 10.1186/s12913-023-10510-x, PMID: 38212786 PMC10782684

[ref190] Telayneh A. T. Gedefaw M. Haile D. Habtegiorgis S. D. Getahun D. S. Tafere Y.. (2021). Cigarette smoking prevalence and associated factors among college students, Amhara, Ethiopia. Pan Afr. Med. J. 40:170. doi: 10.11604/pamj.2021.40.170.24413, PMID: 34970412 PMC8683456

[ref191] Toral V. M. Morales-Domínguez Z. Granado-Alcón M. D. C. Díaz-Milanés D. Andrés-Villas M. (2021). Mediterranean diet, psychological adjustment and health perception in university students: the mediating effect of healthy and unhealthy food groups. Nutrients 13:3769. doi: 10.3390/nu13113769, PMID: 34836022 PMC8621952

[ref192] Tuma F. (2021). The use of educational technology for interactive teaching in lectures. Ann. Med. Surg. 62, 231–235. doi: 10.1016/j.amsu.2021.01.051, PMID: 33537136 PMC7840803

[ref193] Tunc C. G. Bilgin C. N. Cerit B. (2021). The relationship between international students’ health perceptions and their healthy lifestyle behaviors. J. Relig. Health 60, 4331–4344. doi: 10.1007/s10943-021-01336-0, PMID: 34245435 PMC8272445

[ref194] Twizeyimana E. Bihoyiki T. Maniraho J. C. (2021). Overview on teacher-learner insight into computer-assisted teaching and learning: acceptance of powerpoint presentation for enhancing science content visualization. J. Glob. Res. Educ. Soc. Sci. 15, 14–22.

[ref195] Vadivel B. Alam S. Anwar C. Teferi H. (2023). Examining the relationship between antisocial behavior and the academic performance of teenagers: the role of schools and causes of the antisocial behavior. Educ. Res. Int. 2023, 1–11. doi: 10.1155/2023/9463882

[ref196] Valkov P. (2018). School dropout and substance use: consequence or predictor. Trakia J. Sci. 16, 95–101. doi: 10.15547/tjs.2018.02.004

[ref197] van de Groep I. H. Bos M. G. N. Popma A. Crone E. A. Jansen L. M. C. (2023). A neurocognitive model of early onset persistent and desistant antisocial behavior in early adulthood. Front. Hum. Neurosci. 17:1100277. doi: 10.3389/fnhum.2023.1100277, PMID: 37533586 PMC10392129

[ref198] Verma A. Patyal A. Meena J. K. Mathur M. Mathur N. (2021). Interactive teaching in medical education: experiences and barriers. Adesh Univ. J. Med. Sci. Res. 3, 69–73. doi: 10.25259/AUJMSR_13_2021

[ref001] Verma A. K. Singh G. Patwardhan K. (2022). Patterns of physical activity among university students and their perceptions about the curricular content concerned with health: Cross-sectional study. JMIRx Med. 29:e31521. doi: 10.2196/31521 PMC10414421 37725547

[ref199] Villafuerte-Díaz A. Abate M. Moreno C. Ramos P. (2024). Antisocial behaviors at school: analysis of normative and at-risk groups. Child Youth Serv. Rev. 166:107918. doi: 10.1016/j.childyouth.2024.107918

[ref200] Walker S. N. Hill-Polerecky D. M. (1996). Psychometric evaluation of the health-promoting lifestyle profile II, vol. 13 Omaha: University of Nebraska Medical Center, 120–126.

[ref201] Walker S. N. Sechrist K. R. Pender N. J. (1987). The health promoting lifestyle profile: development and psychometric characteristics. Nurs. Res. 36, 76–81. PMID: 3644262

[ref202] Ward S. Williams J. van Ours J. C. (2021). Delinquency, arrest and early school leaving. Oxf. Bull. Econ. Stat. 83, 411–436. doi: 10.1111/obes.12393

[ref203] Waters A. F. Peltier M. R. Roys M. R. Stewart S. A. Copeland A. L. (2021). Smoking and suicidal ideation among college students: smoking expectancies as potential moderators. J. Am. Coll. Heal. 69, 951–958. doi: 10.1080/07448481.2020.1719112, PMID: 32027235 PMC10935596

[ref204] Weiss B. Nguyen T. Trung L. Ngo V. Lau A. (2019). Tobacco smoking and antisocial deviance among Vietnamese, Vietnamese-American, and European-American adolescents. J. Abnorm. Child Psychol. 47, 59–69. doi: 10.1007/s10802-018-0416-8, PMID: 29564575 PMC6150857

[ref205] Whatnall M. C. Patterson A. J. Brookman S. Convery S. Swan C. Pease S.. (2019). Lifestyle behaviors and related health risk factors in a sample of Australian university students. J. Am. Coll. Heal. 68, 734–741. doi: 10.1080/07448481.2019.1611580, PMID: 31140957

[ref206] Wilson O. W. A. Bopp C. M. Bopp M. (2024). Perceived weight change and contributing factors among college students. Int. J. Kinesiol. High. Educ. 8, 63–70. doi: 10.1080/24711616.2023.2209523

[ref207] Wilson O. W. A. Walters S. R. Naylor M. E. Clarke J. C. (2021). Physical activity and associated constraints following the transition from high school to university. Recreat. Sports J. 45, 52–60. doi: 10.1177/1558866121995138

[ref208] Winpenny E. M. Smith M. Penney T. Foubister C. Guagliano J. M. Love R.. (2020). Changes in physical activity, diet, and body weight across the education and employment transitions of early adulthood: a systematic review and meta-analysis. Obes. Rev. 21:e12962. doi: 10.1111/obr.12962, PMID: 31955496 PMC7079102

[ref209] Woo J. G. Zhang N. Fenchel M. Jacobs D. R. Jr. Hu T. Urbina E. M.. (2020). Prediction of adult class II/III obesity from childhood BMI: the i3C consortium. Int. J. Obes. 44, 1164–1172. doi: 10.1038/s41366-019-0461-6, PMID: 31597933 PMC7141944

[ref210] World Health Organization (WHO). The economics of tobacco. (2022). Available at: https://www.euro.who.int/en/health-topics/disease-prevention/tobacco/publications/the-economics-of-tobacco (accessed April 13, 2022).

[ref211] World Health Organization (WHO). Suicide. (2023). Available at: https://www.who.int/news-room/fact-sheets/detail/suicide (accessed May 14, 2024).

[ref212] Worsley J. D. Harrison P. Corcoran R. (2021). Accommodation environments and student mental health in the UK: the role of relational spaces. J. Ment. Health 32, 175–182. doi: 10.1080/09638237.2021.1922648, PMID: 34008464

[ref213] Wu R. Zhu H. Wang Z. J. Jiang C. L. (2021). A large sample survey of suicide risk among university students in China. BMC Psychiatry 21, 1–9. doi: 10.1186/s12888-021-03480-z, PMID: 34583673 PMC8477567

[ref214] Xiang J. Gao J. Gao Y. (2024). The effect of subjective exercise experience on anxiety disorder in university freshmen: the chain-mediated role of self-efficacy and interpersonal relationship. Front. Psychol. 21:1292203. doi: 10.3389/fpsyg.2024.1292203 PMC10914978 38449758

[ref215] Ye B. Ma T. Chen C. Liu M. Wang X. Yang Q. (2022). Exploring the profiles of aggressive behavior among college students: a person-centered approach. Curr. Psychol. 41, 8078–8090. doi: 10.1007/s12144-020-01267-1

[ref216] Younas N. Aftab S. Nisar I. (2023). Comprehensive study of student's criminal activities in educational institutes. J. Posit. Sch. Psychol. 7, 30–40.

[ref217] Young V. J. Burke T. J. Curran M. A. (2019). Interpersonal effects of health-related social control: positive and negative influence, partner health transformations, and relationship quality. J. Soc. Pers. Relat. 36, 3986–4004. doi: 10.1177/0265407519846565

[ref218] Zafar M. S. Nauman M. Nauman H. Nauman S. Kabir A. Shahid Z.. (2021). Impact of stress on human body: a review. Eur. J. Med. Health Sci. 3, 1–7. doi: 10.24018/ejmed.2021.3.3.821

[ref219] Zeng W. Shang S. Fang Q. He S. Li J. Yao Y. (2021). Health promoting lifestyle behaviors and associated predictors among clinical nurses in China: a cross-sectional study. BMC Nurs. 20, 230–211. doi: 10.1186/s12912-021-00752-7, PMID: 34789261 PMC8597212

[ref220] Zengin M. (2021). Investigation of high school students' dropout risk level. Shanlax Int. J. Educ. 9, 59–68. doi: 10.34293/education.v9iS1-May.4000

[ref221] Zhan Y. Wang P. Zhan Y. Lu Z. Guo Y. Ahmad N. A.. (2024). Clustering of lifestyle risk factors in relation to suicidal thoughts and behaviors in young adolescents: a cross-national study of 45 low- and middle-income countries. BMC Glob. Public Health 2:24. doi: 10.1186/s44263-024-00055-4 39681898 PMC11622970

[ref222] Zhang X. Huang P. F. Li B. Q. Xu W. J. Li W. Zhou B. (2021). The influence of interpersonal relationships on school adaptation among Chinese university students during COVID-19 control period: multiple mediating roles of social support and resilience. J. Affect. Disord. 285, 97–104. doi: 10.1016/j.jad.2021.02.040 33640862 PMC9189258

[ref223] Zhang J. Xu L. Li J. Sun L. Qin W. Ding G.. (2019). Gender differences in the association between body mass index and health-related quality of life among adults:a cross-sectional study in Shandong, China. BMC Public Health 19:1021. doi: 10.1186/s12889-019-7351-7, PMID: 31366336 PMC6668122

